# Fibromyalgia and Central Sensitization in Rheumatoid Arthritis, Psoriatic Arthritis, and Spondyloarthritis: A Systematic Review

**DOI:** 10.7759/cureus.113931

**Published:** 2026-08-04

**Authors:** Hassan A Eltoum, Namaa Tilal Osman Hashim, Sagangal Abdelhadi Mohammed, Dania Gamal Hassanelbasri, Asim Ahmed, Marwa A Alamin

**Affiliations:** 1 Internal Medicine and Rheumatology, Omdurman Teaching Hospital, Khartoum, SDN; 2 Allergy and Immunology, Al Kamel General Hospital, Ministry of Health, Makka, SAU; 3 Clinical Immunology, Ahfad University for Women, Khartoum, SDN; 4 General Practice, East Jeddah General Hospital, Jeddah, SAU; 5 Medicne and Surgery, University of Gezira, Wad Madani, SDN; 6 Internal Medicine/Allergy and Clinical Immunology, King Fahd Central Hospital, Jizan, SAU

**Keywords:** central sensitization, fibromyalgia, inflammatory rheumatic diseases, psoriatic arthritis, rheumatoid arthritis

## Abstract

Fibromyalgia (FM) and central sensitization are increasingly recognized as clinically relevant in adults with inflammatory arthritis and spondyloarthritis, particularly when persistent pain and patient-reported symptoms complicate disease activity assessment. This systematic review synthesized clinical evidence on FM and central sensitization in adults with rheumatoid arthritis (RA), psoriatic arthritis (PsA), axial spondyloarthritis (axSpA), ankylosing spondylitis (AS), and other spondyloarthritis (SpA) populations. FM and central sensitization were treated as the primary concepts of interest. Related pain and sensory measures, including nociplastic pain features, neuropathic pain-like symptoms, reduced pressure pain thresholds, and quantitative sensory testing findings, were considered supportive evidence rather than equivalent measures of FM or central sensitization because they reflect different aspects of pain and sensory processing. A total of 47 primary reports met the eligibility criteria after a July 28, 2026 PubMed/MEDLINE update identified 12 additional eligible reports. Assessment approaches included FM diagnostic or classification criteria, FM screening instruments, Central Sensitization Inventory (CSI) measures, quantitative sensory testing, pressure pain thresholds, painDETECT, DN4, and related pain instruments. Across disease groups, FM and higher CSI-defined symptom burden were associated with higher symptom-sensitive composite disease activity scores, greater pain burden, fatigue, sleep disturbance, mood symptoms, impaired physical function, and poorer quality of life. Newer longitudinal and mechanistic studies also showed that pain sensitivity may change with disease-modifying therapy while objective inflammatory and sensory findings do not always move in parallel. Observational treatment-related evidence linked FM or higher symptom burden with difficult-to-manage or treatment-refractory phenotypes, remission classification, treatment retention, and biologic switching, without establishing causal treatment effects. Overall, FM and central sensitization-related symptom burden were frequently reported and were associated with higher symptom-sensitive disease activity scores and poorer patient-reported outcomes. Interpretation was most clinically relevant when symptom burden appeared disproportionate to objective inflammatory findings, while recognizing that active inflammation and amplified pain mechanisms may coexist.

## Introduction and background

Inflammatory rheumatic diseases are chronic immune-mediated conditions in which inflammation, pain, stiffness, fatigue, and impaired physical function can substantially affect daily life and long-term health outcomes. In rheumatoid arthritis (RA), treatment aims to control inflammation, prevent structural damage, and achieve remission or low disease activity through early and targeted use of disease-modifying antirheumatic drugs [1]. In psoriatic arthritis (PsA), assessment is more complex because several domains may be involved, including peripheral arthritis, axial disease, enthesitis, dactylitis, skin disease, pain, fatigue, and quality-of-life impairment [2]. In axial spondyloarthritis (axSpA), treatment decisions also depend on the combined interpretation of symptoms, physical function, inflammatory markers, and imaging findings [3]. These differences highlight the importance of distinguishing active inflammation from other contributors to persistent pain when interpreting disease activity.

Treat-to-target strategies rely on the regular assessment of disease activity and adjustment of treatment toward remission or low disease activity [4]. Depending on the instrument, composite disease activity scores may combine clinician-assessed findings, laboratory markers, and patient-reported components. Pain-sensitive components, such as tender joint count, patient global assessment, fatigue, stiffness, and functional limitation, are clinically meaningful but may also be influenced by factors other than active inflammation. Consequently, a high disease activity score may sometimes reflect both inflammatory activity and additional symptom burden, making persistent symptoms difficult to interpret when objective inflammatory findings are limited.

Fibromyalgia (FM) is a chronic pain syndrome characterized by widespread pain and associated symptoms, such as fatigue, non-restorative sleep, cognitive symptoms, and somatic distress. Revised FM diagnostic criteria emphasize widespread pain distribution and symptom severity rather than tender point examination alone and recognize that FM can be diagnosed in the presence of other clinical disorders [5]. This is particularly relevant in inflammatory arthritis, where pain may arise from active inflammation, structural or mechanical factors, sleep or mood disturbance, altered pain processing, or a combination of these factors. Distinguishing these contributors is important when persistent symptoms appear disproportionate to objective inflammatory findings and when treatment response is being interpreted.

Central sensitization is a neurophysiological mechanism characterized by increased responsiveness within central nociceptive pathways, which may result in increased pain sensitivity, hyperalgesia, allodynia, and enhanced responses to sensory input [6]. Nociplastic pain is related but distinct and describes pain arising from altered nociception when tissue damage or disease of the somatosensory system does not fully explain the pain experience [7]. FM, central sensitization, and nociplastic pain are therefore related but not interchangeable concepts: FM is a clinical syndrome, central sensitization is a neurophysiological mechanism, and nociplastic pain is a pain descriptor associated with altered nociception. In this review, FM and central sensitization were the primary concepts of interest. Neuropathic pain-like symptoms, reduced pressure pain thresholds, quantitative sensory testing findings, and other pain-related measures were considered supportive evidence when they helped characterize altered pain or sensory processing; they were not treated as equivalent diagnoses or direct measures of FM or central sensitization. The Central Sensitization Inventory was likewise treated as a symptom-based inventory rather than a direct physiological measure or diagnostic gold standard for central sensitization.

Previous literature has shown that comorbid FM is common in inflammatory arthritis and may be associated with differences in disease activity scores, patient-reported outcomes, and clinical interpretation across RA, PsA, and axSpA [8]. More recent studies have also examined central sensitization and related pain and sensory features to better understand persistent symptoms that may not correspond closely with inflammatory markers, swollen joint counts, or imaging findings. This broader approach is clinically relevant because altered pain processing and inflammation may coexist, and patients may have clinically important pain-related features without fulfilling formal FM criteria. Misinterpreting such symptom burden as uncontrolled inflammation may complicate clinical assessment and decisions about treatment escalation.

Despite growing interest in this area, important gaps remain. Evidence is distributed across different inflammatory arthritis and spondyloarthritis (SpA) populations, and studies vary considerably in how FM, central sensitization, and related pain features are defined and assessed. Assessment approaches range from FM diagnostic or screening criteria to symptom questionnaires and sensory testing methods. In addition, much of the literature has focused on prevalence or symptom burden, while fewer syntheses have examined disease activity assessment, patient-reported outcomes, discordance with objective inflammation, and treatment-related outcomes together.

A focused synthesis is therefore needed across the inflammatory arthritis and SpA populations represented in the available evidence. This systematic review aimed to synthesize clinical evidence on FM and central sensitization in adults with RA, PsA, and SpA. It examined prevalence and assessment approaches; associations with disease activity and patient-reported outcomes; discordance between clinical disease activity scores and objective inflammatory findings; and relationships with remission, treatment response, retention, escalation, and treatment interpretation.

## Review

Methods

Study Design and Reporting Framework

This systematic review synthesized clinical evidence on FM and central sensitization in adults with RA, PsA, and SpA. FM and central sensitization were the primary concepts of interest. Related pain-mechanism constructs and measures were treated as supportive evidence when they characterized altered pain or sensory processing and were not interpreted as interchangeable diagnoses or direct measures of central sensitization.

The review was reported in accordance with the Preferred Reporting Items for Systematic Reviews and Meta-Analyses (PRISMA) 2020 statement, which guides transparent reporting of systematic reviews, including study identification, screening, eligibility assessment, and synthesis [9].

The protocol was not prospectively registered in PROSPERO and was not published before the review. However, the review question, eligibility criteria, search strategy, data-extraction fields, synthesis approach, and RoB assessment plan were defined before title and abstract screening, full-text assessment, data extraction, and evidence synthesis.

Research Question

This review addressed the following question: In adults with RA, PsA, or SpA, what is the frequency of FM and central sensitization-related findings, and how are these constructs associated with disease activity, patient-reported outcomes, objective inflammation, and treatment-related outcomes?

Because the review included both prevalence-based and association-based evidence, these were interpreted as related but distinct evidence streams. For reporting and synthesis, the primary outcome domains were criterion-based FM prevalence, the frequency of central sensitization-related or screen-positive findings, and their associations with composite disease activity scores. Secondary outcome domains included patient-reported outcomes, objective inflammatory measures, remission, treatment response, treatment retention, treatment escalation, and treatment interpretation.

Related pain-mechanism measures were included only as supportive evidence when they contributed to the interpretation of FM, central sensitization, or altered pain processing. The PECO framework and eligible study designs are shown in Table 1.

**Table 1:** PECO framework and eligible study designs ASDAS, Ankylosing Spondylitis Disease Activity Score; BASDAI, Bath Ankylosing Spondylitis Disease Activity Index; CDAI, Clinical Disease Activity Index; CPDAI, Composite Psoriatic Disease Activity Index; CRP, C-reactive protein; DAPSA, Disease Activity Index for Psoriatic Arthritis; DAS28, Disease Activity Score in 28 joints; DN4, Douleur Neuropathique 4; ESR, erythrocyte sedimentation rate; PASDAS, Psoriatic Arthritis Disease Activity Score; PECO, Population, Exposure, Comparator, Outcome; SDAI, Simplified Disease Activity Index

Component	Definition
Population	Adults aged 18 years or older with rheumatoid arthritis, psoriatic arthritis, axial spondyloarthritis, ankylosing spondylitis, or other spondyloarthritis populations, as defined by the original study criteria. Connective-tissue diseases and other rheumatic conditions outside these disease groups were not eligible.
Exposure	Primary exposures were fibromyalgia, fibromyalgia syndrome, and central sensitization. Related pain and sensory constructs or measures, including central sensitivity syndrome, nociplastic pain, pain sensitization, widespread pain, neuropathic pain-like features, reduced pressure pain thresholds, quantitative sensory testing findings, painDETECT, DN4, and related validated instruments, were included only as supportive measures when they helped characterize altered pain or sensory processing.
Comparator	Patients with the same inflammatory rheumatic disease without fibromyalgia or central sensitization, patients with lower pain sensitization measures, control groups, baseline disease status, or within-study comparison groups where applicable.
Outcomes	Primary outcomes: criterion-based fibromyalgia prevalence; frequency of central sensitization-related or screen-positive findings; and associations of these constructs with composite disease activity scores, including DAS28, CDAI, SDAI, DAPSA, CPDAI, PASDAS, BASDAI, and ASDAS. Secondary outcomes: pain, fatigue, sleep, mood, function, disability, quality of life, patient global assessment, objective inflammatory measures including swollen joint count, ESR, CRP, ultrasound synovitis, and power Doppler activity, and treatment-related outcomes including remission, minimal disease activity, treatment response, biologic switching, treatment retention, treatment escalation, and treatment interpretation.
Study designs	Cross-sectional studies, case-control studies, prospective or retrospective cohort studies, registry-based studies, longitudinal studies, baseline cohort analyses, randomized or interventional feasibility studies, and other primary clinical studies providing extractable relevant data.

Eligibility Criteria

Studies were included if they met all of the following criteria: human clinical studies involving adults aged 18 years or older with RA, PsA, axSpA, AS, or other SpA populations; assessed FM or central sensitization as a primary exposure or reported related pain or sensory measures that supported interpretation of these constructs; reported at least one primary or secondary outcome defined above; provided extractable clinical outcome data; and were published in English. Publication access status was not an eligibility criterion, and studies were not excluded solely because they were subscription-based or otherwise not openly accessible.

The review was restricted to English-language publications because the review team did not have sufficient proficiency in other languages to assess eligibility and extract data reliably. This restriction may have introduced language bias.

Studies were excluded if they were animal or in vitro studies, pediatric-only studies, case reports, editorials, letters, narrative reviews, systematic reviews, or meta-analyses. Connective-tissue diseases, including systemic lupus erythematosus, systemic sclerosis, and idiopathic inflammatory myopathies, and other rheumatic diseases outside the RA, PsA, and SpA groups defined for this review were outside the review scope. Studies were also excluded if they did not assess FM or central sensitization-related features, lacked extractable outcome data, or did not report a relevant primary or secondary outcome defined for this review.

RoB findings were not used as eligibility criteria, and no study was excluded solely on the basis of methodological quality or RoB assessment.

Systematic reviews and meta-analyses were not included as primary evidence to avoid double-counting of participants and overlapping datasets. Such publications were considered only for background context, reference checking, or discussion of existing evidence where appropriate.

Information Sources and Search Strategy

A comprehensive electronic search was conducted across major biomedical databases and supplementary sources. The original search covered records from database inception through August 31, 2025 and included PubMed/MEDLINE, Embase, Scopus, Web of Science, the Cochrane Central Register of Controlled Trials (CENTRAL), and Google Scholar. Reference lists of included studies and relevant reviews were also screened manually to identify additional eligible primary studies.

The original search combined three broad concepts: inflammatory rheumatic diseases; FM, central sensitization, and related pain-processing constructs; and the prespecified outcome domains, including disease activity, patient-reported outcomes, objective inflammation, remission, and treatment response. Supportive search terms included "nociplastic pain", "pain sensitization", "widespread pain", "neuropathic pain-like symptoms", "painDETECT", "DN4", "pressure pain thresholds", and "quantitative sensory testing". Google Scholar results were ranked by relevance, and the first 100 results were screened; the historical rationale for limiting screening to the first 100 relevance-ranked results was not separately documented. Eligibility was restricted to English-language publications for the reason described above.

Records retrieved from the original search sources were exported and managed as a combined library before screening. Because screening was conducted after merging and deduplication, source-specific historical retrieval counts, exact interface-specific search dates, database-platform details, and line-by-line historical search logs were not retained separately and cannot be reconstructed reliably. Accordingly, the retained original strategies are reported as database-adapted search strategies rather than exact historical executable search histories. Records identified through reference-list screening were incorporated into the combined library, and a separate historical count for this route was not retained. The retained search strategies for the original sources are presented in Table 2.

**Table 2:** Search strategies for the original database search and the 2026 PubMed/MEDLINE update The original strategies represent the database-adapted search strings retained from the historical search and should not be interpreted as exact platform-specific historical executable logs. The PubMed/MEDLINE update conducted through the PubMed interface on July 28, 2026, is reported using the exact executable query retained for that search. ASDAS, Ankylosing Spondylitis Disease Activity Score; BASDAI, Bath Ankylosing Spondylitis Disease Activity Index; CDAI, Clinical Disease Activity Index; CENTRAL, Cochrane Central Register of Controlled Trials; CPDAI, Composite Psoriatic Disease Activity Index; CRP, C-reactive protein; CSI, Central Sensitization Inventory; DAPSA, Disease Activity Index for Psoriatic Arthritis; DAS28, Disease Activity Score in 28 joints; DN4, Douleur Neuropathique 4; ESR, erythrocyte sedimentation rate; FiRST, Fibromyalgia Rapid Screening Tool; MeSH, Medical Subject Headings; PASDAS, Psoriatic Arthritis Disease Activity Score; SDAI, Simplified Disease Activity Index; TNF, tumor necrosis factor

Source	Search strategy
PubMed/MEDLINE (original)	("rheumatoid arthritis" OR "psoriatic arthritis" OR "axial spondyloarthritis" OR "ankylosing spondylitis" OR spondyloarthritis OR "inflammatory arthritis" OR "inflammatory rheumatic disease") AND (fibromyalgia OR "fibromyalgia syndrome" OR "central sensitization" OR "central sensitivity syndrome" OR "pain sensitization" OR "nociplastic pain" OR "widespread pain" OR "non-nociceptive pain" OR "neuropathic pain" OR painDETECT OR DN4 OR "pressure pain threshold" OR "quantitative sensory testing" OR "Central Sensitization Inventory" OR CSI OR FiRST OR "Fibromyalgia Rapid Screening Tool") AND ("disease activity" OR DAS28 OR CDAI OR SDAI OR DAPSA OR CPDAI OR PASDAS OR BASDAI OR ASDAS OR remission OR "minimal disease activity" OR "treatment response" OR biologic OR TNF OR "quality of life" OR fatigue OR sleep OR function OR ultrasound OR "power Doppler" OR CRP OR ESR)
Embase (original)	('rheumatoid arthritis' OR 'psoriatic arthritis' OR 'axial spondyloarthritis' OR 'ankylosing spondylitis' OR spondyloarthritis OR 'inflammatory arthritis' OR 'inflammatory rheumatic disease') AND (fibromyalgia OR 'fibromyalgia syndrome' OR 'central sensitization' OR 'central sensitivity syndrome' OR 'pain sensitization' OR 'nociplastic pain' OR 'widespread pain' OR 'non nociceptive pain' OR 'neuropathic pain' OR paindetect OR dn4 OR 'pressure pain threshold' OR 'quantitative sensory testing' OR 'central sensitization inventory' OR csi OR first OR 'fibromyalgia rapid screening tool') AND ('disease activity' OR das28 OR cdai OR sdai OR dapsa OR cpdai OR pasdas OR basdai OR asdas OR remission OR 'minimal disease activity' OR 'treatment response' OR biologic OR tnf OR 'quality of life' OR fatigue OR sleep OR function OR ultrasound OR 'power doppler' OR crp OR esr)
Scopus (original)	TITLE-ABS-KEY(("rheumatoid arthritis" OR "psoriatic arthritis" OR "axial spondyloarthritis" OR "ankylosing spondylitis" OR spondyloarthritis OR "inflammatory arthritis" OR "inflammatory rheumatic disease") AND (fibromyalgia OR "fibromyalgia syndrome" OR "central sensitization" OR "central sensitivity syndrome" OR "pain sensitization" OR "nociplastic pain" OR "widespread pain" OR "non-nociceptive pain" OR "neuropathic pain" OR painDETECT OR DN4 OR "pressure pain threshold" OR "quantitative sensory testing" OR "Central Sensitization Inventory" OR CSI OR FiRST OR "Fibromyalgia Rapid Screening Tool") AND ("disease activity" OR DAS28 OR CDAI OR SDAI OR DAPSA OR CPDAI OR PASDAS OR BASDAI OR ASDAS OR remission OR "minimal disease activity" OR "treatment response" OR biologic OR TNF OR "quality of life" OR fatigue OR sleep OR function OR ultrasound OR "power Doppler" OR CRP OR ESR))
Web of Science (original)	TS=(("rheumatoid arthritis" OR "psoriatic arthritis" OR "axial spondyloarthritis" OR "ankylosing spondylitis" OR spondyloarthritis OR "inflammatory arthritis" OR "inflammatory rheumatic disease") AND (fibromyalgia OR "fibromyalgia syndrome" OR "central sensitization" OR "central sensitivity syndrome" OR "pain sensitization" OR "nociplastic pain" OR "widespread pain" OR "non-nociceptive pain" OR "neuropathic pain" OR painDETECT OR DN4 OR "pressure pain threshold" OR "quantitative sensory testing" OR "Central Sensitization Inventory" OR CSI OR FiRST OR "Fibromyalgia Rapid Screening Tool") AND ("disease activity" OR DAS28 OR CDAI OR SDAI OR DAPSA OR CPDAI OR PASDAS OR BASDAI OR ASDAS OR remission OR "minimal disease activity" OR "treatment response" OR biologic OR TNF OR "quality of life" OR fatigue OR sleep OR function OR ultrasound OR "power Doppler" OR CRP OR ESR))
CENTRAL (original)	("rheumatoid arthritis" OR "psoriatic arthritis" OR "axial spondyloarthritis" OR "ankylosing spondylitis" OR spondyloarthritis OR "inflammatory arthritis") AND (fibromyalgia OR "central sensitization" OR "central sensitivity syndrome" OR "pain sensitization" OR "nociplastic pain" OR "widespread pain") AND ("disease activity" OR remission OR "treatment response" OR biologic OR TNF OR "quality of life")
Google Scholar (original)	Documented core terms: "fibromyalgia" "central sensitization" "rheumatoid arthritis" "psoriatic arthritis" "axial spondyloarthritis" "disease activity" DAS28 DAPSA BASDAI ASDAS "quality of life" "treatment response". Results were ranked by relevance, and the first 100 results were screened.
PubMed/MEDLINE update (July 28, 2026)	("Arthritis, Rheumatoid"[MeSH Terms] OR "rheumatoid arthritis"[Title/Abstract] OR "Arthritis, Psoriatic"[MeSH Terms] OR "psoriatic arthritis"[Title/Abstract] OR "Spondylitis, Ankylosing"[MeSH Terms] OR "ankylosing spondylitis"[Title/Abstract] OR "axial spondyloarthritis"[Title/Abstract] OR "axSpA"[Title/Abstract] OR "Spondylarthritis"[MeSH Terms] OR "Spondylarthropathies"[MeSH Terms] OR spondyloarthritis[Title/Abstract]) AND ("Fibromyalgia"[MeSH Terms] OR fibromyalgia[Title/Abstract] OR "fibromyalgia syndrome"[Title/Abstract] OR "central sensitization"[Title/Abstract] OR "central sensitivity syndrome"[Title/Abstract] OR "Central Sensitization Inventory"[Title/Abstract] OR nociplastic[Title/Abstract] OR "pain sensitization"[Title/Abstract] OR "pressure pain threshold"[Title/Abstract] OR "pressure pain thresholds"[Title/Abstract] OR "quantitative sensory testing"[Title/Abstract] OR painDETECT[Title/Abstract] OR "neuropathic pain-like"[Title/Abstract]) AND ("2025/09/01"[Date - Publication] : "3000"[Date - Publication])

To update the evidence base, an additional PubMed/MEDLINE search was conducted through the PubMed interface on July 28, 2026, covering publications from September 1, 2025 onward. The update used a broader two-concept strategy combining inflammatory rheumatic disease terms with FM, central sensitization, and related pain-construct terms, without requiring an outcome-related search block. The search retrieved 159 records, which were screened using the same eligibility criteria as the original review. The update was documented separately from the historical search rather than being retrospectively incorporated into the original source-specific retrieval counts. Unlike the retained original strategies, the complete executable query for the July 28, 2026 PubMed/MEDLINE update was preserved and is provided in Table 2.

Record Management and Deduplication

All retrieved records were imported into Zotero for reference management. Duplicate records were identified using Zotero's duplicate-detection function and removed before screening. The remaining records were exported to Microsoft Excel for screening documentation and manual verification. Additional duplicate checking in Excel used article title, author names, publication year, journal name, and DOI when available.

Study Selection

Hassan A. A. Eltoum (HAAE) and Namaa Tilal Osman Hashim (NTOH) independently screened titles and abstracts against the eligibility criteria described above. Records that were clearly irrelevant were excluded at this stage. Full texts of potentially relevant reports were then retrieved and independently assessed by the same two reviewers.

Studies were selected if they included adults with RA, PsA, axSpA, AS, or other SpA populations and reported FM, central sensitization, or related pain or sensory measures relevant to these constructs. Eligible studies were also required to report at least one relevant primary or secondary outcome defined for this review.

Disagreements between reviewers were resolved through discussion until consensus was reached. Primary reasons for full-text exclusion were documented during study selection; however, the original study-level exclusion log was not retained in a form that allowed reliable retrospective category-specific counts. Reports that could not be obtained were recorded as not retrieved rather than excluded on scientific eligibility grounds. No formal quantitative measure of reviewer agreement was calculated. The study selection process was documented using a PRISMA 2020 flow diagram [9].

Handling of Multiple or Overlapping Reports

When multiple reports appeared to use overlapping or related study populations, they were compared using author names, study setting, recruitment period, eligibility criteria, disease group, exposure definition, comparator, sample size, and reported outcomes. Reports were treated as separate publications only when they addressed distinct outcomes, follow-up periods, or substudy questions. Where cohorts overlapped, report-level findings were retained when relevant, but the same participant population was not counted as independent evidence within the same outcome synthesis.

When systematic reviews or meta-analyses were identified, they were not included as primary evidence. However, their reference lists were checked to identify potentially eligible primary studies.

Data Items and Extraction

Data extraction was performed independently by Hassan A. A. Eltoum (HAAE) and Namaa Tilal Osman Hashim (NTOH) using a structured Microsoft Excel extraction form. Extracted variables included first author, publication year, country, study design, disease group, participant characteristics, sample size, FM or central sensitization assessment tool, diagnostic or screening criteria, related pain-mechanism measures, criterion-based FM prevalence and the frequency of central sensitization-related or screen-positive findings where reported, comparator group, disease activity measures, patient-reported outcomes, objective inflammatory measures, treatment-related outcomes, main findings, and study conclusion.

Where available, extracted outcome data included prevalence values, group differences, baseline values, follow-up values, measures of variance, 95% confidence intervals, p-values, and units of measurement. For treatment-response studies, between-group or responder-based comparisons were prioritized over within-group changes when available. For observational studies, adjusted estimates were prioritized over unadjusted estimates when reported.

The two reviewers cross-checked extracted data for completeness and accuracy, and inconsistencies were resolved through discussion and consensus. No formal quantitative agreement statistic was calculated, and no separate formal calibration exercise was documented. Study authors were not contacted for missing or unclear information. When relevant data could not be verified from the available report, the information was recorded as not reported and numerical values were not imputed.

Extracted outcomes included criterion-based FM prevalence; central sensitization-related or screen-positive findings; disease activity scores, pain intensity, fatigue, sleep disturbance, mood symptoms, physical function, disability, quality of life, patient global assessment, tender joint count, swollen joint count, erythrocyte sedimentation rate (ESR), C-reactive protein (CRP), ultrasound synovitis, power Doppler activity, remission, minimal disease activity, treatment response, biologic switching, treatment retention, treatment escalation, and treatment interpretation.

Outcome Domains

For synthesis, outcomes were grouped into predefined domains. Frequency outcomes included criterion-based FM, FM screening-positive findings, and study-reported central sensitization-related symptom burden. Central sensitivity syndrome and related pain-mechanism indicators, including nociplastic pain, pain sensitization, widespread pain, non-nociceptive pain, neuropathic pain-like symptoms, reduced pressure pain thresholds, quantitative sensory testing abnormalities, painDETECT, DN4, and related measures, were interpreted as supportive evidence when they helped characterize altered pain or sensory processing.

Assessment-tool data included the criteria or instruments used to classify or screen for FM and to assess central sensitization-related symptom burden or other pain phenotypes, including the American College of Rheumatology FM criteria, Fibromyalgia Survey Diagnostic Criteria, Fibromyalgia Rapid Screening Tool, Fibromyalgia Assessment Screening Tool 4, Central Sensitization Inventory, quantitative sensory testing, pressure pain thresholds, painDETECT, DN4, tender point counts, and related measures.

Disease activity outcomes included DAS28, DAS28-ESR, DAS28-CRP, CDAI, SDAI, DAPSA, CPDAI, PASDAS, BASDAI, ASDAS-CRP, ASDAS-ESR, BASFI, and related composite indices. Patient-reported outcomes included pain, fatigue, sleep, mood, depression, anxiety, patient global assessment, physical function, disability, health-related quality of life, work-related outcomes, and patient acceptable symptom state.

Objective inflammation outcomes included swollen joint count, ESR, CRP, musculoskeletal ultrasound, grey-scale ultrasound synovitis, power Doppler activity, and other objective inflammatory measures. Treatment-related outcomes included remission, minimal disease activity, treatment response, TNF inhibitor response, TNF inhibitor retention, biologic switching, DMARD decision-making, treatment escalation, and treatment misclassification.

Study Risk-of-Bias Assessment

RoB was assessed using tools selected according to the actual design and outcome contribution of each study rather than relying only on the design label used by the original authors. Analytical cross-sectional studies were assessed using the Joanna Briggs Institute (JBI) Critical Appraisal Checklist for Analytical Cross-Sectional Studies, while prevalence-focused cross-sectional studies were assessed using the JBI Checklist for Studies Reporting Prevalence Data [10]. Baseline-only analyses from cohort studies were treated as cross-sectional for the outcome of interest when no longitudinal information contributed to that analysis.

Cohort, registry-based, longitudinal, and case-control evidence contributing longitudinal or comparative outcomes was assessed using the Newcastle-Ottawa Scale, considering selection, comparability, and outcome or exposure domains [11]. The randomized feasibility study was assessed using the revised Cochrane RoB tool for randomized trials (RoB 2) at the relevant outcome level, covering bias arising from the randomization process, deviations from intended interventions, missing outcome data, measurement of outcomes, and selection of the reported result [12]. The study design, outcome contribution, and appraisal tool applied to each study were mapped explicitly in the RoB reporting.

RoB assessment was conducted independently by the two reviewers, with disagreements resolved through discussion and consensus. JBI checklists and the Newcastle-Ottawa Scale were interpreted using their tool-specific items or domains and were not converted into a common four-category low/moderate/high/critical scale. RoB 2 judgments followed the categories native to RoB 2. Internal RoB concerns were distinguished from imprecision or applicability issues; factors such as small sample size, absence of healthy controls, or limited generalizability were not used by themselves to determine internal RoB. Tool-specific bias-relevant concerns were summarized transparently in the RoB presentation. RoB findings were used to interpret the evidence and were not used to exclude studies.

Synthesis Methods

Before deciding whether to pool results quantitatively, the feasibility of meta-analysis was assessed in a structured manner according to disease group, construct, diagnostic or assessment definition, instrument and cutoff, outcome, effect measure, follow-up period, study design, and independence of the underlying cohort. FM, central sensitization-related symptom burden, and supportive pain or sensory measures were evaluated as distinct constructs and were not combined in a single quantitative estimate. Quantitative pooling was considered only when studies were sufficiently comparable across these domains and reported compatible numerical data.

Meta-analysis was not undertaken when meaningful comparability could not be established without combining non-equivalent constructs, definitions, instruments, denominators, effect measures, or overlapping populations. Prevalence findings based on FM diagnostic or classification criteria were considered separately from FM screening-positive findings and from Central Sensitization Inventory-defined symptom burden. Quantitative sensory testing, pressure pain thresholds, painDETECT, DN4, and related supportive measures were synthesized separately according to the question each instrument addressed. For proportions with an explicit numerator and denominator that were not fixed by study design, descriptive 95% confidence intervals were calculated using the Wilson score method for tabular reporting; these estimates were not pooled. Confidence intervals were not derived when an explicit numerator was unavailable or when a reported percentage could not be reconciled with the stated denominator.

The narrative synthesis followed structured principles consistent with Synthesis Without Meta-analysis (SWiM). Studies were grouped first by disease population and then by construct and outcome domain. Effect estimates were reported using the metric provided by each study. Where both adjusted and unadjusted association estimates were available, adjusted estimates were prioritized. Direction of effect was interpreted consistently within each outcome domain rather than across non-equivalent measures.

Multiple outcomes or models from the same study were treated as correlated findings from one report rather than as independent pieces of evidence. When several adjusted models were reported for the same outcome, the estimate from the most fully adjusted clinically relevant model was prioritized when clearly identifiable; otherwise, the range or pattern of reported estimates was summarized without selecting a single model as definitive. Statistical significance alone was not used to determine consistency across studies.

Findings were synthesized narratively by disease group and outcome domain, including study characteristics, FM prevalence, central sensitization or screen-positive symptom burden, supportive pain or sensory findings, disease activity scores, patient-reported outcomes, discordance between clinical disease activity and objective inflammation, treatment response, remission, treatment retention or switching, treatment interpretation, and RoB. Within each domain, heterogeneity was assessed qualitatively by comparing populations, definitions and instruments, effect metrics, direction and magnitude of associations, follow-up, analytical adjustment, and RoB findings.

Cross-sectional findings were interpreted as associations rather than evidence of causation. Longitudinal and registry-based findings were used to describe temporal or treatment-related patterns but were interpreted with attention to confounding and other observational limitations. Randomized feasibility evidence was interpreted at the relevant outcome level and cautiously in view of its feasibility design. Where possible, findings were also considered in relation to disease type, inflammatory marker profile, objective imaging, treatment exposure, disease activity score components, patient-reported burden, and remission or treatment-response definitions.

Certainty of Evidence

Formal certainty-of-evidence grading using GRADE was not performed. Therefore, conclusions were based on a qualitative assessment of consistency of findings, study design, RoB, directness of outcomes, sample size, clinical relevance of outcomes, and agreement between clinical scores, patient-reported outcomes, and objective inflammatory measures. The absence of formal GRADE certainty assessment is acknowledged as a limitation.

Results

Study Selection and PRISMA Flow

**Figure 1:**
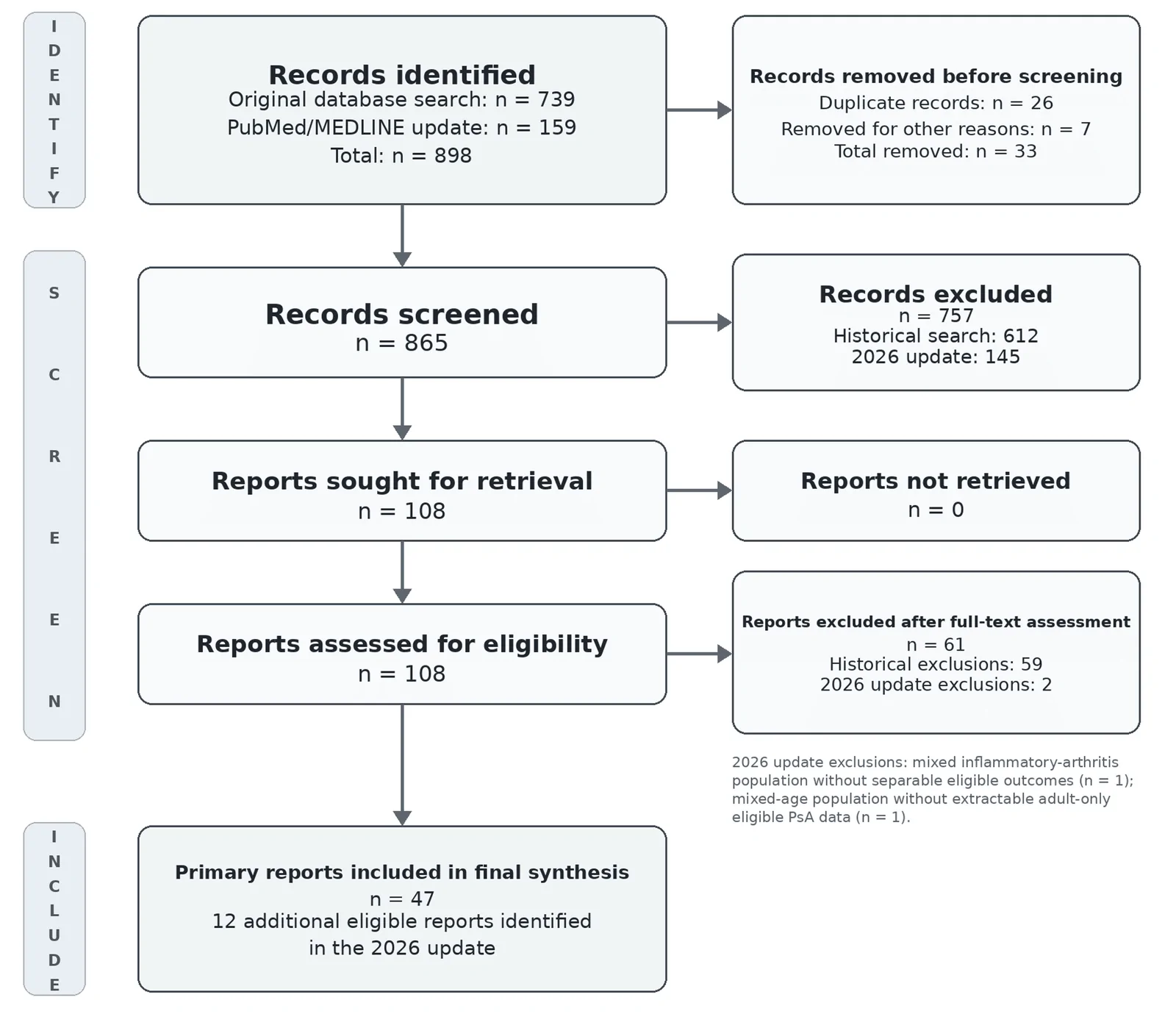
PRISMA 2020 flow diagram of the study selection The diagram combines the preserved historical search totals with the separately documented July 28, 2026 PubMed/MEDLINE update. Source-specific historical retrieval counts and category-specific reasons for the 59 historical full-text exclusions were not retained and were not retrospectively reconstructed. PRISMA, Preferred Reporting Items for Systematic Reviews and Meta-Analyses

The original search identified 739 records, and the July 28, 2026 PubMed/MEDLINE update identified 159 additional records, giving 898 records overall. Before screening, 26 duplicate records and seven records removed for other reasons from the historical search set were excluded, leaving 865 records for title and abstract screening. Of these, 757 were excluded (612 from the historical search and 145 from the update), and 108 reports were sought for retrieval and assessed for eligibility; all 108 were retrieved. Following full-text assessment, 61 reports were excluded. The historical exclusion log supported the verified total of 59 reports that did not meet the eligibility criteria but did not support reliable retrospective numerical subcategories. In the update, one report was excluded because a mixed inflammatory-arthritis population included noneligible participants and did not provide separable outcomes for the prespecified disease groups, and one was excluded because a mixed-age population did not provide extractable adult-only data for the eligible PsA subgroup. The final synthesis therefore included 47 eligible primary reports, of which 12 were added by the 2026 update. The study-selection process is summarized in Figure 1.

Study Characteristics

The final synthesis included 47 eligible primary reports published between 2004 and 2026 and examining FM, central sensitization-related symptom burden, and related pain phenotypes in adults with RA, PsA, axSpA, AS, and other SpA populations [13-59]. The term report is used because related publications from overlapping cohorts were retained only when they contributed distinct outcomes, follow-up periods, or substudy questions. Related pain-mechanism measures, including nociplastic pain features, pain sensitization, neuropathic pain-like symptoms, pressure pain thresholds, quantitative sensory testing, and other pain-sensitivity measures, were retained only when they supported interpretation of FM or central sensitization-related symptom burden; they were not treated as interchangeable constructs.

RA remained the most frequently represented disease group. The 2026 update added six reports containing eligible RA data [48,53-55,57,58], four reports containing PsA-specific data [50,51,56,57], and six reports containing axSpA, AS, or other SpA data [49,50,52,54,57,59]; some reports contributed to more than one eligible disease group. Potential overlap between the Salamanca PsA reports by Chacón et al. and Llamas-Ramos et al. was considered when interpreting shared outcomes [51,56]. van der Kraan et al. explicitly reported a secondary analysis of the GLAS cohort and was not treated as an independent population for outcomes overlapping with prior GLAS reports [59]. Most reports were cross-sectional; the remainder included cohort, registry-based, longitudinal, case-control, and randomized feasibility designs.

FM was identified using American College of Rheumatology (ACR) classification or diagnostic criteria, Fibromyalgia Survey Diagnostic Criteria, study-defined diagnostic approaches, or screening instruments. FiRST and FAST 4 findings are reported as screen-positive when no diagnostic assessment was performed. CSI cutoff findings are reported as screen-positive or high CSI symptom burden rather than as diagnostic evidence of central sensitization. Quantitative sensory testing, pressure pain thresholds, painDETECT, DN4, and related measures were treated as purpose-specific supportive measures. The study characteristics and extractable results are summarized in Table 3.

**Table 3:** Characteristics and principal findings of the included primary reports Percentages and 95% confidence intervals were calculated from explicit numerator/denominator data; otherwise, values are shown as reported. Deliberately selected comparison groups were not treated as prevalence samples. CSI cutoff results indicate screen positivity rather than diagnostic central sensitization. AS, ankylosing spondylitis; ASAS, Assessment of SpondyloArthritis International Society; b/tsDMARD, biologic/targeted synthetic disease-modifying antirheumatic drug; CASPAR, Classification Criteria for Psoriatic Arthritis; CPM, conditioned pain modulation; CRP, C-reactive protein; D2M, difficult-to-manage; DMARD, disease-modifying antirheumatic drug; EGA, evaluator global assessment; ESR, erythrocyte sedimentation rate; EULAR, European Alliance of Associations for Rheumatology; FACIT-F, Functional Assessment of Chronic Illness Therapy-Fatigue; FIQ, Fibromyalgia Impact Questionnaire; FMF, familial Mediterranean fever; GS-US7, seven-joint grey-scale ultrasound score; HADS, Hospital Anxiety and Depression Scale; nr-axSpA, nonradiographic axial spondyloarthritis; NRS, numeric rating scale; OR, odds ratio; PASI, Psoriasis Area Severity Index; PASS, Patient Acceptable Symptom State; PD-US7, seven-joint power Doppler ultrasound score; SJC, swollen joint count; TJC, tender joint count; TS, temporal summation

Study	Design / population / sample	Fibromyalgia (FM), central sensitization (CS), or supportive assessment and frequency	Comparator / follow-up / adjustment	Primary outcome(s)	Key quantitative finding(s)
Joharatnam N et al., 2015 [13]	UK, cross-sectional; rheumatoid arthritis (RA), n = 50.	American College of Rheumatology (ACR) 2010 FM criteria: 24/50 (48.0%; 95% confidence interval (CI) 34.8-61.5); pressure pain threshold (PPT) supportive measure.	FM-classified vs. non-FM comparisons, continuous Widespread Pain Index (WPI)+Symptom Severity Scale (SSS) symptom-score analyses, and PPT analyses; cross-sectional; multivariable models adjusted for age and gender.	Disease Activity Score in 28 joints using erythrocyte sedimentation rate (DAS28-ESR), pressure pain thresholds, mental health	patient-reported proportion of the Disease Activity Score in 28 joints (DAS28-P) was associated with FM classification after adjustment (adjusted odds ratio (aOR) 2.8, 95% CI 1.2-6.4; p = 0.014) and with WPI+SSS score (standardized beta = 0.50; p = 0.001). More sensitive PPTs were linked to higher patient-reported Disease Activity Score in 28 joints (DAS28) components.
Brikman S et al., 2016 [14]	Israel, cross-sectional; psoriatic arthritis (PsA), n = 73.	ACR 1990/2010 FM criteria: 13/73 (17.8%; 95% CI 10.7-28.1).	Cross-sectional comparison; no longitudinal follow-up; adjusted effect estimate not reported.	Composite Psoriatic Disease Activity Index (CPDAI), Disease Activity Index for Psoriatic Arthritis (DAPSA), DAS28, minimal disease activity (MDA), Health Assessment Questionnaire (HAQ), quality of life (QoL)	CPDAI 9.23 ± 1.92 vs. 4.25 ± 3.14 (p < 0.001); DAPSA 27.53 ± 19.23 vs. 12.82 ± 12.71 (p = 0.003); MDA 0% vs. 43.3% (p = 0.003).
Salaffi F et al., 2024 [15]	Italy; cross-sectional; PsA without coexisting FM, n=157.	Central Sensitization Inventory (CSI) ≥40: 45.2% screen-positive as reported (n=157; explicit numerator not reported); interpreted as high CSI symptom burden rather than diagnostic CS.	No follow-up; multivariable model assessed independent contribution to 12-item Psoriatic Arthritis Impact of Disease (PsAID-12).	DAPSA, Psoriatic Arthritis Disease Activity Score (PASDAS), PsAID-12, HAQ, QoL	CSI correlations: DAPSA rho = 0.587; PASDAS rho = 0.573; HAQ rho = 0.607; 36-Item Short Form Health Survey (SF-36) physical rho = -0.405 and mental rho = -0.483. CSI independently contributed to PsAID-12 (p < 0.001).
Öz N et al., 2025 [16]	Türkiye, cross-sectional; axial spondyloarthritis (axSpA) on biologic therapy for ≥6 months, n=120.	CSI ≥40: 49/120 (40.8%; 95% CI 32.5-49.8) screen-positive.	CSI screen-positive vs. screen-negative; no follow-up; backward multivariable logistic regression evaluated predictors of CSI ≥40.	Bath Ankylosing Spondylitis Disease Activity Index (BASDAI), Ankylosing Spondylitis Disease Activity Score using C-reactive protein (ASDAS-CRP), Ankylosing Spondylitis Quality of Life (ASQoL)	ASQoL (OR 3.030, 95% CI 1.755-5.230; p < 0.001) and BASDAI (OR 2.221, 95% CI 1.134-4.350; p = 0.020) independently predicted CSI ≥40. ASQoL area under the curve (AUC)=0.97 (95% CI 0.95-0.99).
Macfarlane GJ et al., 2018 [17]	UK, prospective national register; axSpA, n=1,757; tumor necrosis factor inhibitor (TNFi) starters included in response analysis, n=291.	2011 FM research criteria: 388/1757 (22.1%; 95% CI 20.2-24.1).	FM criteria met vs. not met; multivariable linear regression adjusted for demographic, clinical, and lifestyle factors; TNFi response followed to 12 months.	BASDAI, Ankylosing Spondylitis Disease Activity Score (ASDAS), ASQoL, TNFi response	Adjusted BASDAI difference 1.04 (95% CI 0.75-1.33); ASQoL difference 1.42 (0.88-1.96). No significant difference in Assessment of SpondyloArthritis International Society 20% response (ASAS20) at 12 months.
Şaş S et al., 2023 [18]	Turkey, cross-sectional; axSpA, n = 116; controls, n = 95.	CSI ≥40: 54/116 (46.6%; 95% CI 37.7-55.6) screen-positive; ACR 2010 FM criteria: 28/116 (24.1%; 95% CI 17.3-32.7); Douleur Neuropathique 4 (DN4) ≥4: 27/116 (23.3%; 95% CI 16.5-31.7). Controls: CSI 13/95 (13.7%), FM 6/95 (6.3%), DN4 5/95 (5.3%).	axSpA vs. age- and sex-matched controls and within-axSpA CSI comparisons; cross-sectional; no longitudinal follow-up.	BASDAI, ASDAS-CRP, Bath Ankylosing Spondylitis Functional Index (BASFI), ASQoL	CSI screen positivity was 46.6% in axSpA vs. 13.7% in controls; FM 24.1% vs. 6.3%; DN4-positive neuropathic pain-like features 23.3% vs. 5.3% (all p < 0.001). CSI screen-positive patients had higher disease activity and worse function/QoL.
Wolfe F et al., 2004 [19]	USA, national self-report survey; RA, n = 11,866.	Survey-defined FM characteristics: Regional Pain Scale ≥8 plus fatigue visual analogue scale (VAS) ≥6; 1,731 patients (17.1%, as reported by the authors). The reported percentage could not be reconciled with the total cohort denominator (n=11,866), so no recalculated CI was added.	Cross-sectional survey comparison; no longitudinal follow-up; adjusted effect estimates reported for selected outcomes.	Rheumatoid Arthritis Disease Activity Index (RADAI), HAQ, QoL, costs, disability	RA with FM had worse symptoms, poorer function, lower QoL utility, more comorbidities, and higher medical costs.
Ahmed L et al., 2024 [20]	UK, open-label randomized feasibility trial; RA, n = 25.	Supportive pain-mechanism measures only: painDETECT, quantitative sensory testing (QST) and VAS; no FM/CS diagnostic frequency reported.	Randomized groups; quarterly assessments over 12 months; feasibility study, not powered for definitive comparative efficacy.	Disease Activity Score in 28 joints using C-reactive protein (DAS28-CRP), pain modalities during biologic therapy	VAS pain improvement over 12 months, reported as mean and standard error of the mean (SEM), was 3.7 (0.82) with abatacept and 2.3 (1.1) with adalimumab; painDETECT and QST improved in both groups.
Alp G et al., 2025 [21]	Türkiye, single-center cross-sectional; CASPAR-classified PsA, n=114.	CSI ≥40: 49/114 (43.0%; 95% CI 34.3-52.2) screen-positive; DN4 ≥4: 24/102 (23.5%; 95% CI 16.4-32.6); ACR 2016 fibromyalgia syndrome (FMS): 28/110 (25.5%; 95% CI 18.2-34.3).	CSI screen-positive vs. screen-negative and DN4-positive vs. negative comparisons; cross-sectional; multivariable analysis identified factors independently associated with CSI screen positivity.	DAPSA, MDA, Health Assessment Questionnaire Disability Index (HAQ-DI), mood outcomes	CSI screen positivity was associated with higher pain, global assessments, TJC, DAPSA, and HAQ-DI. Anxiety, depression, and HAQ-DI were independently associated with CSI screen positivity; body mass index (BMI) and FMS were associated with DN4-positive neuropathic pain-like features.
Kaya MN et al., 2023 [22]	Turkey; single-center cross-sectional observational study; axSpA n=30, FMF n=30, axSpA/FMF n=30, healthy controls n=30; total n=120.	CSI Part A (0-100) assessed symptom burden and severity; no single binary CSI screen-positive prevalence was reported. Mean CSI Part A in axSpA was 41.0 ± 19.8 versus 15.0 ± 9.4 in healthy controls.	Four-group cross-sectional comparison; no longitudinal follow-up. Analyses compared CSI scores and biopsychosocial outcomes across disease groups and controls.	CSI Part A, ASDAS, BASDAI, quality of life, sleep, anxiety, depression	CSI Part A scores were higher in axSpA, FMF, and axSpA/FMF than in healthy controls (p < 0.001), with no significant difference among the three disease groups. No FM/CS prevalence was inferred from CSI Part A.
Bello N et al., 2016 [23]	France, cross-sectional clinical cohort; clinician-diagnosed spondyloarthritis (SpA), n=196; 185/196 (94.4%) met ASAS classification criteria.	Fibromyalgia Rapid Screening Tool (FiRST) ≥5/6: 42/196 (21.4%; 95% CI 16.3-27.7) screen-positive; not diagnostic FM.	FiRST screen-positive vs. screen-negative; historical TNFi exposure/retention analyzed with Kaplan-Meier and Cox regression; first-TNFi retention assessed to 2 years.	BASDAI, BASFI, TNFi retention	First-TNFi retention at 2 years was 28.1% (95% CI 12.5-44.0) vs. 41.7% (95% CI 32.2-51.3; p < 0.01). Switchers were more frequent in the FiRST-positive group (15.2% vs. 3.8%; p = 0.03); TNFi exposure itself did not differ significantly.
Coury F et al., 2009 [24]	France; cross-sectional outpatient comparison; 182 women total: RA n=105, RA+FM n=49, FM-only n=28; RA population n=154.	ACR 1990 FM criteria: 49/154 RA patients (31.8%; 95% CI 25.0-39.5).	RA+FM vs. RA and FM-only groups; cross-sectional; regression analysis of DAS28 components; no longitudinal follow-up.	DAS28, HAQ, SF-36, radiographic joint damage	RA+FM had higher clinical disease activity than RA alone; regression analysis showed different weighting of DAS28 components. The study did not support interpreting the association as a marker of worse structural prognosis.
Dhir V et al., 2009 [25]	North India, cross-sectional; RA, n = 200; controls, n = 200.	ACR 1990 FM criteria: 30/200 (15.0%; 95% CI 10.7-20.6); controls 2.5%.	Cross-sectional comparison; no longitudinal follow-up; adjusted effect estimate not reported.	three-component Disease Activity Score in 28 joints (DAS28-3), HAQ-DI, pain, fatigue, sleep	FM was associated with higher DAS28-3, worse HAQ-DI, more pain, fatigue, and sleep disturbance.
Ton E et al., 2012 [26]	Netherlands; cross-sectional outpatient study; 200 recruited, 196 eligible RA patients.	Tender-point count (supportive, not FM diagnosis): ≥11 points 15%; 6-10 points 12%; 1-5 points 30%; 0 points 43% among 196 eligible patients.	Follow-up/adjustment details not reported.	DAS28 and DAS28 components	Tender-point burden affected DAS28, TJC and patient global health, but not SJC or ESR; no FM prevalence was inferred.
Nawito Z et al., 2013 [27]	Egypt; selected cross-sectional comparison; 50 women with RA (25 RA+FM; 25 RA without FM) drawn from 150 screened patients.	ACR FM criteria; 25 RA+FM and 25 RA without FM were deliberately selected. The 25/50 split is not a prevalence estimate.	Cross-sectional comparison; no longitudinal follow-up; adjusted effect estimate not reported.	DAS28, Clinical Disease Activity Index (CDAI), Modified Health Assessment Questionnaire (MHAQ)	DAS28 5.6 ± 1.1 vs. 4.5 ± 1.3 (p = 0.009); CDAI 23.3 ± 12.1 vs. 13.7 ± 11.0 (p = 0.002); MHAQ 0.7 ± 0.6 vs. 0.31 ± 0.4 (p = 0.006).
Zammurrad S et al., 2013 [28]	Pakistan, comparative cross-sectional; ACR/EULAR 2010-classified RA, n=138.	ACR 1990 FM criteria: 31/138 (22.4% as reported; 95% CI 16.3-30.1).	Cross-sectional comparison; no longitudinal follow-up; adjusted effect estimate not reported.	DAS28	Mean DAS28 5.3 ± 1.5 with FM vs. 3.9 ± 1.2 without FM (p < 0.001).
da Silva Chakr RM et al., 2015 [29]	Brazil, matched case-control; RA, n = 72 women.	ACR 2010 FM criteria; matched 36 RA+FM and 36 RA without FM. The 1:1 split is design-imposed and not a prevalence estimate.	Cross-sectional comparison; no longitudinal follow-up; adjusted effect estimate not reported.	DAS28, Simplified Disease Activity Index (SDAI), CDAI, GS-US7, PD-US7	RA with FM had higher clinical disease activity scores but similar ultrasound synovitis scores.
Ghib LJ et al., 2015 [30]	Romania, case-control; RA, n = 30.	RA by ACR/EULAR 2010; FM by ACR 1990; selected groups: RA+FM n=10, RA n=10, FM n=10; not prevalence sampling.	Cross-sectional comparison; no longitudinal follow-up; adjusted effect estimate not reported.	DAS28, CDAI, ultrasound	Mean DAS28 5.6 vs. 4.6 vs. 4.5 (RA+FM, RA, FM); RA+FM and RA had similar ultrasound scores.
Mian AN et al., 2016 [31]	UK, cross-sectional; active RA with DAS28 >2.6, n=47.	TJC-SJC ≥7 (fibromyalgic joint-count criterion): 26/47 (55.3%; 95% CI 41.2-68.6); ACR 1990 FM tender-point criterion: 19/47 (40.4%; 95% CI 27.6-54.7); both criteria: 17/47 (36.2%; 95% CI 24.0-50.5).	Criterion-positive vs. criterion-negative groups; cross-sectional; blinded hand/wrist ultrasound; no longitudinal follow-up.	DAS28, grey-scale ultrasound, power Doppler ultrasound, pain, fatigue, disability, mood	Patients meeting both criteria had lower grey-scale ultrasound scores (15.4 vs. 21.8; p = 0.022) and lower power Doppler scores (2.94 vs. 8.33; p = 0.028) despite higher clinical disease activity.
da Silva Chakr RM et al., 2017 [32]	Brazil, prospective cohort; RA, n = 256.	ACR 1990 FM criteria: 32/256 (12.5%; 95% CI 9.0-17.1).	Mean follow-up 6.2 ± 2.0 years (2,986 visits); observational treatment-decision analyses.	DAS28, HAQ, DMARD decisions	Predefined treatment-misclassification scenarios at 28.4% of visits with FM vs. 19.8% without FM (p < 0.001); no causal treatment effect inferred.
Salaffi F et al., 2017 [33]	Italy, observational longitudinal cohort; Long-standing RA, n = 117.	Concomitant FM: 20/117 (17.1%; 95% CI 11.3-24.9).	Six-month follow-up; logistic regression included comorbidity burden, FM and SF-36 mental component.	SDAI remission	24/117 (20.4%) achieved SDAI remission overall; none with concomitant FM achieved remission. FM associated with non-remission (p = 0.0001); exact OR not reported.
Durmaz Y et al., 2021 [34]	Turkey, retrospective cross-sectional; RA, n = 381.	ACR 2016 FM criteria: 98/381 (25.7%; 95% CI 21.6-30.3).	Cross-sectional comparison; no longitudinal follow-up; adjusted effect estimate not reported.	DAS28 versions, biologic switching	RA with FM had higher tender joint counts and DAS28 scores and switched treatment more often due to perceived non-response.
Saitou M et al., 2022 [35]	Japan, cross-sectional; RA, n = 240.	CSI ≥40: 18/240 (7.5%; 95% CI 4.8-11.5) screen-positive for central sensitivity syndrome (CSS); CSI is a screening questionnaire, not a diagnostic test for CS.	CSI severity/screen-positive comparisons; cross-sectional; multivariable analyses examined correlates of CSS and neuropathic pain-like symptoms.	DAS28-CRP, DAS28-ESR, CDAI, SDAI, painDETECT, HADS, SF-36	CSS was associated with EGA (OR 0.860), fibromyalgia symptom scale (OR 1.46), painDETECT (OR 1.24), anxiety (OR 1.35), and SF-36 physical (OR 0.898), mental (OR 0.828), and role-social (OR 0.946) scores. CSI score contributed most to neuropathic pain-like symptoms (beta = 0.266; p < 0.001).
Sami M et al., 2023 [36]	Egypt, cross-sectional; Active RA, n = 100.	Study-defined secondary FM: 67/100 (67.0%; 95% CI 57.3-75.4); FM ascertainment criterion not reported.	Cross-sectional comparison; no longitudinal follow-up; adjusted effect estimate not reported.	DAS28, CDAI, musculoskeletal ultrasound (MSUS)	DAS28 4.99 ± 0.82 vs. 4.22 ± 0.96 and CDAI 30.49 ± 10.59 vs. 18.0 ± 10.68 (both p < 0.001); MSUS did not differ significantly.
El-Kasmi H et al., 2024 [37]	Morocco, observational cross-sectional; ACR/EULAR-classified RA, n=97.	Fibromyalgia Assessment Screening Tool (FAST 4) ≥3/4: 30/97 (30.9%; 95% CI 22.6-40.7) screen-positive; FiRST screen-positive 30.4%.	No follow-up; logistic regression assessed associated factors.	DAS28-ESR, depression/anxiety	FAST 4 sensitivity 78.6%, specificity 87.1% vs. FiRST. Depression independently associated with FAST 4 positivity: OR 5.917 (95% CI 1.91-18.3), p = 0.002.
Mesci N et al., 2024 [38]	Türkiye, cross-sectional; ACR/EULAR 2010-classified RA, n=60.	CSI ≥40: 29/60 (48.3%; 95% CI 36.2-60.7) screen-positive; ACR 1990 FMS: 20/60 (33.3%; 95% CI 22.7-45.9).	Cross-sectional comparison; no longitudinal follow-up; adjusted effect estimate not reported.	DAS28-ESR, DAS28-CRP, PPT, QoL	CSI screen-positive status was associated with higher VAS pain, TJC, and DAS28 and lower PPT, while SJC, ESR, and CRP were comparable.
Navarini L et al., 2024 [39]	Italy, multicenter cross-sectional; PsA, n = 221.	Revised ACR 2016 FM criteria; concomitant FM 36.53% as reported.	No follow-up; multivariable logistic models adjusted for concomitant FM.	DAPSA, MDA, CPDAI, BASDAI, PASS	After FM adjustment, PASS was not significantly associated with DAPSA low disease activity: OR 0.514 (95% CI 0.215-1.227), p = 0.134.
Kaya MN et al., 2024 [40]	Türkiye, single-center cross-sectional; CASPAR-classified PsA, n=103.	CSI ≥40: 67/103 (65.1% as reported; 95% CI 55.4-73.6) screen-positive.	CSI screen-positive vs. screen-negative; no follow-up; multivariable logistic regression included clinical and psychosocial variables.	DAS28, DAPSA, QoL, sleep, function	PASI was the strongest reported predictor of CSI screen positivity (OR 9.70, 95% CI 1.52-62.21); CSI screen-positive patients had worse clinical and functional outcomes.
Kieskamp SC et al., 2022 [41]	Netherlands, cross-sectional analysis of the Groningen Leeuwarden Axial Spondyloarthritis (GLAS) cohort; axSpA, n = 178.	CSI ≥40: 80/178 (44.9%; 95% CI 37.8-52.3) screen-positive; reported as 45%, indicating a high probability of CS-related symptom burden.	Cross-sectional baseline analysis; multivariable linear regression adjusted for ASDAS-CRP, sex, entheseal involvement, comorbidities, symptom duration, smoking, BMI class, and education.	ASDAS-CRP, BASDAI, ASQoL	CSI score remained associated with ASQoL after adjustment: B = 0.04 (95% CI 0.03-0.05); univariable B = 0.06 (0.05-0.07).
Mogard E et al., 2021 [42]	Sweden, cross-sectional analysis of the population-based SPARTAKUS cohort; axSpA n=175 (AS n=120; nr-axSpA n=55).	Supportive pain-sensitivity assessment (cuff pressure algometry: threshold, tolerance, temporal summation); no FM/CS diagnostic frequency.	AS vs. nr-axSpA and linear regression analyses at a single assessment; no longitudinal treatment effect evaluated.	ASDAS-CRP, BASDAI, BASFI	Lower pain tolerance was associated with worse disease-activity/function measures; no FM or CS prevalence inferred from algometry.
Macfarlane GJ et al., 2019 [43]	UK, national register cluster analysis; axSpA, n = 1338.	2011 FM research criteria: 307/1338 (23.0% as reported; 95% CI 20.8-25.3).	Cross-sectional baseline cluster analysis using development and validation split samples; no longitudinal follow-up applicable.	ASDAS, BASDAI, function, QoL, work outcomes	Four baseline clusters were identified. More than half of participants in both high-disease-activity clusters met FM research criteria; lower-activity clusters had substantially lower FM frequency.
Wach J et al., 2016 [44]	France, single-center cross-sectional; SpA, n = 103.	ACR 1990 FM criteria: 18/103 (17.5%; 95% CI 11.3-25.9).	Cross-sectional comparison; no longitudinal follow-up; adjusted effect estimate not reported.	BASDAI, ASDAS-CRP, QoL	Median BASDAI 4.2 vs. 2.2 (p = 0.0068); ASDAS-CRP 2.7 vs. 2.0 was not statistically significant (p = 0.1264).
Gao R, 2021 [45]	China; baseline cross-sectional comparison with short longitudinal follow-up; SpA n=121 (axSpA n=111; peripheral SpA n=10).	ACR 2016 FM criteria: axSpA 11/111 (9.9%; 95% CI 5.6-16.9); peripheral SpA 1/10 (10.0%); overall 12/121 (9.9%; 95% CI 5.8-16.5).	Baseline FM vs. non-FM comparisons; among 107 radiographic axSpA patients, 37 withdrew and 70 were followed for 2 months. Short follow-up observations are non-randomized and non-causal.	ASDAS-CRP, Ankylosing Spondylitis Disease Activity Score using erythrocyte sedimentation rate (ASDAS-ESR), BASDAI, BASFI	At baseline, FM was associated with higher ASDAS, BASDAI, BASFI, ESR, and mood symptom scores. Among 70 followed radiographic axSpA patients, disease-activity/function scores declined over 2 months in both groups, with larger observed declines in the FM group; causal inference is not possible.
Provan SA et al., 2021 [46]	UK, prospective national register; axSpA, n = 801.	Self-reported Fibromyalgia Survey Diagnostic Criteria (2015 version): 115/801 (14.4%; 95% CI 12.1-17.0) at baseline.	Longitudinal FM classification transitions with repeated assessments; multivariable logistic models examined development and loss of FM classification. Associations are observational and non-causal.	BASDAI, ASDAS-CRP, TNFi response	FM development: BASDAI OR 1.27 (95% CI 1.08-1.49), WPI OR 1.14 (1.02-1.28). Loss of FM classification: BASFI OR 0.68 (0.53-0.86), WPI OR 0.84 (0.72-0.97), TNFi initiation OR 3.86 (1.54-9.71).
Kieskamp SC et al., 2025 [47]	Netherlands, cross-sectional structural equation modeling study; axSpA, n = 332.	CSI used as a continuous symptom measure; diagnostic prevalence not reported.	Cross-sectional structural equation model; no longitudinal follow-up. The model included illness perception, psychological and lifestyle factors, BMI, CSI, and ASDAS; causal direction cannot be established.	ASDAS, illness perception, psychosocial factors	In the structural equation model, CSI was positively associated with ASDAS: B = 0.24, standardized beta = 0.24 (95% CI 0.01-0.47), p = 0.040. Illness perception and BMI were also directly associated with ASDAS; the cross-sectional model does not establish causality.
Aydemir B et al., 2025 [48]	USA, prospective 12-week observational analysis; established active RA initiating or switching DMARD therapy, n = 182.	Supportive QST only: PPT at multiple sites, temporal summation, and conditioned pain modulation; no FM/CS diagnostic frequency.	Baseline vs. 12 weeks after DMARD change; adjusted models included age, sex, seropositivity, RA symptom duration, BMI, DMARD class, and study site.	DAS28-CRP, QST/PPT, SJC, TJC, CRP, patient global assessment	DAS28-CRP improved by -1.04 (95% CI -1.30 to -0.77). PPT improved at wrists +0.39 (0.04 to 0.73), thumbnails +0.51 (0.11 to 0.91), and trapezius +0.38 (0.03 to 0.73). Adjusted change in DAS28-CRP was associated with change in PPT at wrists beta = -0.34, knees beta = -0.47, and thumbnails beta = -0.34; TS and CPM did not significantly change.
Bertsias A et al., 2026 [49]	Greece, prospective single-center cohort; axSpA starting targeted agents, n = 434.	FM recorded as a comorbidity/predictor; no criterion-based FM prevalence estimate used for synthesis.	D2M vs. non-D2M; long-term cohort with 2,312 patient-years; multivariable logistic models and comorbidity clustering.	D2M axSpA, function, adverse events/hospitalization	D2M was reported in 50/434 (11.5%). FM was independently associated with D2M (OR 3.522, 95% CI 1.666-7.448; p = 0.001). A chronic-pain comorbidity cluster including FM and osteoarthritis was also associated with D2M (adjusted OR 2.542, 95% CI 1.250-5.171).
Carli L et al., 2026 [50]	Italy, cross-sectional consecutive cohort; PsA n = 79 and AS n = 21; total n = 100.	CSI ≥40: 42/100 (42.0%; 95% CI 32.8-51.8) screen-positive; interpreted as high CSI symptom burden, not diagnostic CS.	Cross-sectional CSI screen-positive vs. screen-negative comparisons; no longitudinal follow-up.	Disease Activity Index for Psoriatic Arthritis using C-reactive protein (DAPSA-CRP), ASDAS, multi-failure status, HAQ, FACIT-F, HADS, SF-36	CSI ≥40 was associated with DAPSA-CRP (p = 0.02), ASDAS (p = 0.03), multi-failure status (p = 0.01), and FM (p = 0.004). CSI also showed significant associations with HADS, FACIT-F, HAQ, and SF-36 domains (p < 0.0001).
Chacón C et al., 2026 [51]	Spain, single-center cross-sectional observational study; consecutive PsA excluding FM, n = 228.	CSI ≥40: 35.1% screen-positive as reported; FM systematically excluded using modified ACR 2016 criteria.	CSI screen-positive vs. screen-negative; no follow-up; binary and linear multivariable regression. Potential cohort overlap with Llamas-Ramos et al. [56].	Clinical DAPSA, ASDAS-CRP, HAQ-DI, painDETECT, sleep, mood, therapeutic refractoriness	CSI screen-positive patients had higher clinical DAPSA (14 vs. 9.8; p = 0.001), ASDAS-CRP (4.8 vs. 1.8; p = 0.007), HAQ-DI (0.8 vs. 0.2; p = 0.001), and therapeutic refractoriness (61.5% vs. 6.8%; p = 0.003). Depression, poor sleep, and painDETECT were independent correlates of CSI burden.
Remalante-Rayco P et al., 2026 [52]	Canada, cross-sectional analysis within a longitudinal axSpA cohort, n = 536.	FM ever recorded as a comorbidity; no criterion-based FM prevalence estimate used for synthesis.	D2M vs. non-D2M and treatment-refractory (TR) vs. non-TR; multivariable bias-reduced logistic regression with multiple imputation and prespecified covariates.	D2M/TR axSpA, spinal pain, inflammatory and non-inflammatory contributors	D2M was present in 49/536 (9.1%) and TR axSpA in 12/536 (2.2%). FM ever was associated with D2M in univariable analysis (OR 5.00, 95% CI 2.50-9.71) and after adjustment (OR 3.09, 95% CI 1.33-7.15; p = 0.009).
Akar ZA et al., 2026 [53]	Türkiye, cross-sectional; RA, n = 160.	PainDETECT-defined likely nociplastic-like pain features: 36/160 (22.5%); supportive pain phenotype rather than diagnostic central sensitization.	Cross-sectional comparisons and stepwise multivariable linear regression; no follow-up.	DAS28, VAS pain, fatigue, sleep, SF-36, CRP, ESR	PainDETECT correlated with pain intensity (r = 0.650) and DAS28 (r = 0.527), with weaker correlations with ESR (r = 0.235) and CRP (r = 0.150). Pain intensity, tender joint count, and education level were retained in the final stepwise model, whereas CRP, ESR, and DAS28 were not retained.
Caglayan M et al., 2026 [54]	Türkiye, cross-sectional; SpA n = 218 and RA n = 142; total n = 360, ages 18-80 years.	ACR 1990 FM criteria: SpA 53/218 (24.3%; 95% CI 19.1-30.4); RA 35/142 (24.6%; 95% CI 18.3-32.3).	Cross-sectional treatment-stratified comparisons; b/tsDMARD exposure was not randomized.	FM frequency, DAS28, BASDAI, FIQ, treatment exposure	FM frequency did not differ between RA and SpA (p = 0.942). Overall, FM was less frequent among b/tsDMARD-treated than untreated participants (20.3% vs. 30.1%; p = 0.033); the association was significant in RA (p = 0.030) but not SpA (p = 0.314).
Çiftci İnceoğlu S et al., 2025 [55]	Türkiye, cross-sectional; RA with hand pain n = 50 and hand osteoarthritis n = 50.	DN4 and painDETECT supportive neuropathic-like pain measures; no FM/CS diagnostic frequency reported.	Single-visit RA vs. hand-osteoarthritis comparison; adjusted effect estimate not reported.	Neuropathic-like symptoms, hand function, VAS pain	VAS, DN4, and painDETECT scores did not differ significantly between RA and hand osteoarthritis (all p > 0.05). Duruöz Hand Index values were worse in RA (p = 0.041).
Llamas-Ramos R et al., 2026 [56]	Spain, single-center cross-sectional; consecutive adult CASPAR-classified PsA, n = 246.	CSI Part A used for central sensitization-related symptom burden; CSI ≥40 defined screen-positive status. FM by 2016 criteria: 16/246 (6.5%; 95% CI 4.0-10.3).	Kinesiophobia-positive vs. negative; multivariable logistic and linear regression. Potential cohort overlap with Chacón et al. [51].	CSI, Clinical Disease Activity Index for Psoriatic Arthritis (cDAPSA), HAQ-DI, sleep, mood, fatigue, kinesiophobia	Kinesiophobia was present in 112/246 (45.5%) and associated with higher CSI (41.5 vs. 29; p < 0.001) and cDAPSA (13 vs. 11; p = 0.001). CSI (OR 1.03, 95% CI 1.00-1.05; p = 0.02) and poor sleep (OR 1.09, 95% CI 1.00-1.10; p = 0.03) were independent correlates.
Petzinna SM et al., 2025 [57]	Germany, exploratory cross-sectional QST study; RA n = 20, PsA n = 20, axSpA n = 20, systemic sclerosis (SSc) n = 20, and controls n = 20. Eligible disease-group results were separable.	Standardized German Research Network on Neuropathic Pain (DFNS) QST; supportive sensory phenotype, not diagnostic FM/CS. Pathological allodynia was reported in RA 15%, PsA 25%, axSpA 15%, and controls 0%.	Disease groups vs. matched controls; single visit; no longitudinal follow-up.	mechanical detection threshold (MDT), vibration detection threshold (VDT), thermal/mechanical sensory measures, disease activity, inflammatory markers	Compared with controls, MDT differed in RA (beta = 0.90; p = 0.025), PsA (beta = 1.30; p = 0.001), and axSpA (beta = 0.80; p = 0.045); VDT also differed in all three eligible groups. QST abnormalities were not associated with disease activity, duration, or inflammatory markers.
Pontes de Oliveira MNGR et al., 2025 [58]	Brazil, descriptive cross-sectional convenience sample; RA, n = 113.	CSI, painDETECT, and revised 2016 FM criteria. CSI screen-positive/high symptom burden: 59.29% as reported. FM criteria were applied only among CSI ≥40 participants.	Cross-sectional descriptive and predictive analyses; no follow-up.	CSI, FM criteria, painDETECT, CDAI, pain NRS	Among CSI screen-positive patients, 47.76% met FM criteria and 52.24% did not. Probable neuropathic-like pain by painDETECT was reported in 17.70% overall. CSI correlated with CDAI (r = 0.446) and pain (r = 0.443) and remained a predictor of CDAI (p = 0.009) and NRS pain (p = 0.037) in multivariable analyses.
van der Kraan YM et al., 2026 [59]	Netherlands, exploratory cross-sectional secondary GLAS analysis; axSpA, n = 201 (128 men, 73 women).	CSI continuous symptom burden plus QST/PPT, temporal summation, and conditioned pain modulation; no independent diagnostic prevalence estimate.	Sex-stratified cross-sectional comparisons and univariable regression; explicitly secondary analysis of a previously published GLAS cohort.	BASDAI pain questions, CSI, PPT, TS, CPM, psychosocial measures	Women had higher CSI scores than men (41.5 ± 13.6 vs. 30.6 ± 13.8; effect size 0.79), lower PPTs, and different pain-related psychosocial profiles. Findings were treated as secondary GLAS evidence and not as an independent cohort for overlapping outcomes.

Prevalence and Screening Findings

Criterion-based FM: In RA, criterion-based FM frequencies varied across clinical samples: 12.5% [32], 15.0% [25], 22.4% [28], 25.7% [34], 31.8% [24], 40.4% in a selected active-RA sample [31], and 48.0% [13]. A selected active-RA cohort reported secondary FM in 67% of participants, although the exact ascertainment criterion was not reported [36]. The update added a 2026 study using ACR 1990 criteria in which FM was present in 35/142 RA patients (24.6%; 95% CI 18.3-32.3) [54]. In PsA, criterion-based FM was reported in 17.8% [14], 25.5% of evaluable patients [21], and 36.53% [39]; Llamas-Ramos et al. additionally identified FM using 2016 criteria in 16/246 PsA patients (6.5%; 95% CI 4.0-10.3) [56]. In axSpA and other SpA populations, criterion-based frequencies included 22.1% and 23.0% using the 2011 research criteria [17,43], 24.1% using the ACR 2010 criteria [18], 17.5% using the ACR 1990 criteria [44], approximately 9.9% using the ACR 2016 criteria [45], and 14.4% at baseline using the self-reported Fibromyalgia Survey Diagnostic Criteria [46]. Caglayan et al. reported ACR 1990 FM in 53/218 SpA patients (24.3%; 95% CI 19.1-30.4) [54]. These estimates were not combined because populations and ascertainment methods differed.

FM screening-positive findings: FAST 4 screening was positive in 30.9% of one RA cohort [37]. In SpA, FiRST screening was positive in 21.4% of one cohort [23]. These values represent screening-positive status rather than criterion-based FM diagnosis or classification.

Central Sensitization Inventory findings: CSI ≥40 was screen-positive in 7.5% [35] and 48.3% [38] of two RA samples; the update added a tertiary-care RA cohort in which 59.29% were CSI screen-positive as reported [58]. In PsA, corresponding findings were 45.2% in a cohort excluding coexisting FM [15], 43.0% [21], and 65.1% [40]. Chacón et al. reported 35.1% CSI screen positivity in 228 PsA patients after systematic exclusion of FM [51]. In mixed PsA/AS populations, Carli et al. reported CSI ≥40 in 42/100 participants (42.0%; 95% CI 32.8-51.8) [50]. In axSpA, previously reported frequencies included 40.8% [16], 46.6% [18], and approximately 45% [41]. Kaya et al. [22], Llamas-Ramos et al. [56], and van der Kraan et al. [59] also contributed continuous CSI findings rather than an independent diagnostic prevalence estimate. CSI findings were interpreted as symptom-burden or screen-positive findings, not as diagnostic evidence of central sensitization.

Supportive pain and sensory phenotypes: Tender-point counts, pressure pain thresholds, quantitative sensory testing, painDETECT, DN4, and related measures were summarized according to their intended construct and were not converted into FM or central sensitization prevalence estimates. The update added longitudinal QST evidence in RA showing improved pressure pain thresholds after 12 weeks of DMARD therapy [48]; a cross-sectional RA study reporting painDETECT-defined nociplastic-like features in 22.5% of patients [53]; a hand-pain study comparing DN4 and painDETECT findings in RA and hand osteoarthritis [55]; standardized QST findings across separable RA, PsA, and axSpA groups [57]; and a GLAS secondary analysis combining CSI with PPT, temporal summation, and conditioned pain modulation [59]. Detailed study-level findings are provided in Table 3.

Effect on Disease Activity Assessment

Across RA reports, FM or CSI-defined symptom burden was frequently associated with higher composite disease activity scores, particularly indices containing pain-sensitive or patient-reported components [13,24-28,34,38,53,58]. Joharatnam et al. found that FM classification and greater fibromyalgianess were independently associated with a higher patient-reported contribution to DAS28 after adjustment for age and sex [13]. Nawito et al. reported higher DAS28 and CDAI in a selected RA-plus-FM group, with the difference attributed mainly to tender joint count and patient global assessment rather than swollen joint count or ESR [27]. Mesci et al. similarly found higher tender joint count and DAS28 among CSI screen-positive patients while swollen joint count, ESR, and CRP were comparable [38]. In the update, PainDETECT scores correlated with DAS28 but only weakly with CRP and ESR in Akar et al. [53], while Pontes de Oliveira et al. reported a positive association between CSI and CDAI [58]. Aydemir et al. provided longitudinal supportive evidence: pressure pain thresholds improved over 12 weeks after DMARD initiation or switching, and reductions in DAS28-CRP were associated with increases in several PPT measures after adjustment [48].

In PsA, FM or CSI screen-positive status was associated with higher composite disease activity and worse patient-reported outcomes in several reports [14,15,21,40,51,56]. Brikman et al. reported higher CPDAI and DAPSA in patients with FM, and no patients with FM met minimal disease activity criteria [14]. Salaffi et al. reported correlations between the CSI score and DAPSA, PASDAS, function, and health-related quality of life [15]. Alp et al. and Kaya et al. also reported higher disease activity and worse functional outcomes among CSI screen-positive patients [21,40]. In 2026, Chacón et al. reported higher clinical DAPSA and ASDAS-CRP values among CSI screen-positive PsA patients [51], while Llamas-Ramos et al. found higher CSI scores and cDAPSA among patients with clinically significant kinesiophobia [56]. Navarini et al. found that the relationship between the patient acceptable symptom state and DAPSA was not significant after adjustment for concomitant FM [39].

In axSpA, AS, and other SpA populations, FM or higher CSI symptom burden was generally associated with higher patient-reported disease activity and poorer function or quality of life [16-18,22,23,41,43-47,49,50,52,54,59]. Macfarlane et al. reported an adjusted mean BASDAI difference of 1.04 points (95% CI 0.75-1.33) and an ASQoL difference of 1.42 points (95% CI 0.88-1.96) for participants meeting FM criteria [17]. Carli et al. found CSI ≥40 associated with DAPSA-CRP or ASDAS according to disease group [50]. Caglayan et al. found nearly identical ACR 1990 FM frequencies in RA and SpA but higher symptom scores within some FM-positive treatment strata [54]. van der Kraan et al., in a GLAS secondary analysis, reported higher CSI values and greater pain sensitivity in women than men with axSpA [59]. Not every composite measure differed consistently; Wach et al., for example, reported higher BASDAI with FM while the ASDAS-CRP difference was not statistically significant [44].

Patient-Reported Outcomes, Function, Sleep, and Quality of Life

Several reports described worse patient-reported outcomes among patients with FM or higher central sensitization-related symptom burden. In RA, Wolfe et al. reported worse symptoms, function, quality-of-life utility, disability, comorbidity burden, and medical costs among patients meeting survey-defined FM criteria [19]. Coury et al. and Dhir et al. reported worse functional status and symptom burden among RA patients with FM [24,25]. Saitou et al. found CSI-defined symptom burden associated with neuropathic pain-like symptoms, anxiety, and poorer SF-36 domains [35]. The update reinforced this pattern: Akar et al. linked higher painDETECT scores to greater pain, fatigue, poorer sleep, and worse SF-36 domains [53], and Pontes de Oliveira et al. linked CSI and painDETECT scores to pain and CDAI [58].

In PsA, CSI screen-positive findings and concomitant FM were associated with worse pain, function, sleep, mood, or quality-of-life outcomes across several reports [15,21,39,40,51,56]. Salaffi et al. found that CSI score correlated with HAQ and both physical and mental components of the SF-36 [15]. Alp et al. reported greater pain and worse HAQ-DI among CSI screen-positive patients [21]. Chacón et al. reported greater functional disability and markedly higher neuropathic pain-like features among CSI screen-positive patients, with depression and poor sleep among the independent correlates [51]. Llamas-Ramos et al. found that higher CSI scores and poorer sleep independently related to kinesiophobia in PsA [56].

In axSpA and other SpA populations, FM and higher CSI symptom burden were associated with poorer function and quality of life [16-18,22,41,43-45,47,49,50,52,59]. Macfarlane et al. reported poorer ASQoL among participants meeting FM criteria [17]. Kieskamp et al. found that CSI score remained associated with ASQoL after adjustment for ASDAS-CRP and demographic and clinical covariates [41]. Carli et al. reported broad associations between CSI and HADS, FACIT-F, HAQ, and SF-36 domains [50]. Difficult-to-manage axSpA cohorts reported greater functional burden in association with FM as a comorbidity [49,52], while van der Kraan et al. demonstrated sex-related differences in CSI, pain sensitivity, and psychosocial profiles [59].

Discordance Between Clinical Disease Activity Scores and Objective Inflammation

Several RA studies compared clinical disease activity with imaging or other objective inflammatory measures. In matched or selected comparisons, patients with FM had higher DAS28, CDAI, or SDAI scores without proportionately greater ultrasound synovitis [29-31,36]. da Silva Chakr et al. reported higher clinical activity scores but similar grey-scale and power Doppler ultrasound scores in RA patients with and without FM [29]. Ghib et al. found a higher mean DAS28 in the RA-plus-FM group but similar ultrasound scores to the RA group [30]. Mian et al. reported that participants meeting both fibromyalgic joint-count and classification criteria had higher clinical disease activity but lower ultrasound inflammation [31].

The same clinical-objective discordance was supported by reports in which pain-sensitive components differed more strongly than inflammatory measures. Nawito et al. reported higher DAS28 and CDAI in a selected RA-plus-FM group, driven mainly by tender joint count and patient global assessment, while objective components were comparable [27]. Mesci et al. reported higher VAS pain, tender joint count, and DAS28 among CSI screen-positive patients despite comparable swollen joint count, ESR, and CRP [38]. Ton et al., using tender-point count as a supportive pain-sensitivity measure rather than an FM diagnosis, found that tender-point burden affected DAS28, tender joint count, and patient global health but not swollen joint count or ESR [26]. Newer mechanistic evidence was directionally consistent but heterogeneous: Akar et al. found substantially stronger correlations of painDETECT with pain and DAS28 than with CRP or ESR [53]; Petzinna et al. found QST-detected sensory alterations in RA, PsA, and axSpA without association with disease activity or inflammatory markers [57]; and Aydemir et al. found improvement in PPTs alongside improvement in DAS28-CRP, although changes in CRP itself were not associated with PPT changes [48]. These patterns support careful interpretation of discordance rather than a binary inflammatory-versus-centralized classification.

Treatment Response, Remission, and Treatment Interpretation

Treatment-related findings were observational or exploratory and were interpreted cautiously. In axSpA, Macfarlane et al. found higher baseline BASDAI and poorer quality of life among participants meeting the FM criteria, but no significant difference in ASAS20 response at 12 months after TNFi initiation [17]. Bello et al. observed lower first-TNFi retention among FiRST screen-positive patients [23], while FM classification changed over time in Provan et al. [46]. The update added difficult-to-manage axSpA evidence: FM was independently associated with D2M status in Bertsias et al. [49] and Remalante-Rayco et al. [52], but these were observational associations and do not establish that FM causes refractory inflammatory disease. Carli et al. also found CSI ≥40 associated with a multi-failure treatment history in a mixed SpA cohort [50].

In RA and PsA, FM or higher symptom burden was associated with remission classification, treatment-decision patterns, or apparent treatment refractoriness, but causal effects were not demonstrated. da Silva Chakr et al. reported more follow-up visits meeting predefined treatment-misclassification scenarios among RA patients with FM [32], Salaffi et al. found no SDAI remission among patients with concomitant FM [33], and Durmaz et al. reported more frequent biologic switching in patients with FM [34]. In the update, Caglayan et al. reported lower FM frequency among b/tsDMARD-treated than untreated participants overall and within RA, but treatment exposure was not randomized, and the cross-sectional association should not be interpreted as a treatment effect [54]. In PsA, Chacón et al. found CSI screen-positive status strongly associated with therapeutic refractoriness, while explicitly acknowledging uncertain temporal and causal direction [51]. Aydemir et al. showed that pressure pain thresholds can improve alongside disease activity after DMARD changes, whereas temporal summation and conditioned pain modulation did not significantly change over 12 weeks [48].

RoB Assessment

RoB was interpreted using the design-appropriate appraisal instrument rather than a common cross-tool severity scale. JBI, Newcastle-Ottawa Scale (NOS), and RoB 2 findings were therefore not converted into unified low/moderate/high categories. Bias-relevant issues included selection and recruitment, confounding, outcome or exposure measurement, overlapping populations, and attrition where applicable. Small samples, single-center settings, restricted subgroup sizes, and limited generalizability were treated primarily as precision or applicability issues unless they also created a specific internal-validity concern. Screening instruments were not considered inherently biased; their construct limitations were reflected in interpretation. RoB 2 was applied at the relevant outcome level for the randomized feasibility study. RoB findings were used for interpretation only and did not determine report inclusion. Table 4 provides a report-specific narrative summary and separates internal-validity concerns from precision, applicability, construct-interpretation, and overlap limitations.

**Table 4:** Risk-of-bias appraisal summary of the included primary reports JBI and NOS appraisals are presented using tool-specific items or domains without mapping them to a common overall category; RoB 2 is interpreted at the relevant outcome level. JBI, Joanna Briggs Institute; JBI Analytical Cross Sectional Checklist, Critical Appraisal Checklist for Analytical Cross Sectional Studies; JBI Prevalence Data Checklist, Checklist for Studies Reporting Prevalence Data; NOS, Newcastle-Ottawa Scale; RoB 2, revised Cochrane Risk of Bias tool for randomized trials

Study	Design / tool	Bias-relevant appraisal	Precision / applicability / interpretive limitation
Joharatnam N et al., 2015 [13]	Cross-sectional / JBI Analytical Cross Sectional Checklist	No specific internal-validity concern was identified from the reported study methods.	Small sample size limited generalizability.
Brikman S et al., 2016 [14]	Cross-sectional / JBI Analytical Cross Sectional Checklist	No specific internal-validity concern was identified from the reported study methods.	Small sample size and single-center setting may limit external validity.
Salaffi F et al., 2024 [15]	Cross-sectional / JBI Analytical Cross Sectional Checklist	Cross-sectional associations and the multivariable model do not establish temporality; residual confounding remains possible.	The study intentionally excluded coexisting FM, so its CSI findings apply to PsA patients without FM. CSI ≥40 is a screening cutoff, not diagnostic evidence of CS.
Öz N et al., 2025 [16]	Cross-sectional / JBI Analytical Cross Sectional Checklist	Cross-sectional associations and logistic-regression predictors do not establish temporal or causal relationships; residual confounding remains possible.	Exclusion of patients with a prior FM diagnosis limits applicability to the broader axSpA population and may influence the observed CSI screening frequency.
Macfarlane GJ et al., 2018 [17]	Registry cohort / Newcastle-Ottawa Scale (NOS)	Attrition at 12-month follow-up may have affected biologic-treatment outcome estimates.	No additional major precision or applicability limitation was identified from the reported study features.
Şaş S et al., 2023 [18]	Cross-sectional / JBI Analytical Cross Sectional Checklist	Controls were recruited from hospital visitors rather than a population-based source, which may affect selection and group comparability.	This limitation is most relevant to patient-versus-control comparisons; within-axSpA associations are less dependent on the control sample.
Wolfe F et al., 2004 [19]	Survey cross-sectional / JBI Checklist for Studies Reporting Prevalence Data	Reliance on self-reported survey data may have introduced information bias.	No additional major precision or applicability limitation was identified from the reported study features.
Ahmed L et al., 2024 [20]	Randomized feasibility trial / RoB 2 (revised Cochrane Risk of Bias tool)	RoB 2 was applied at the relevant outcome level. The open-label design is particularly relevant to subjective pain outcomes because participants and clinicians were aware of treatment allocation.	Very small feasibility sample (n=25) limits precision and the trial was not powered for definitive comparative efficacy; no single study-level RoB category is assigned.
Alp G et al., 2025 [21]	Cross-sectional / JBI Analytical Cross Sectional Checklist	Cross-sectional associations cannot establish temporality. Multivariable analyses identify variables independently associated with CSI screen positivity but do not establish causal risk factors.	Single-center recruitment limits external applicability; FMS and DN4 analyses had smaller evaluable denominators than the full cohort.
Kaya MN et al., 2023 [22]	Cross-sectional / JBI Analytical Cross Sectional Checklist	Single-center cross-sectional group comparisons are susceptible to selection differences and residual confounding; temporality cannot be established.	Each disease/control group contained 30 participants, limiting subgroup precision. CSI Part A was analyzed as symptom severity and is not diagnostic evidence of CS.
Bello N et al., 2016 [23]	Cross-sectional / JBI Analytical Cross Sectional Checklist	Cross-sectional clinical comparisons and observational TNFi retention analyses are susceptible to residual confounding; retention differences do not establish a causal effect of FM.	FiRST defines screen-positive FM status rather than diagnostic FM. Of 196 clinician-diagnosed SpA patients, 185 met ASAS classification criteria.
Coury F et al., 2009 [24]	Cross-sectional comparative study / JBI Analytical Cross Sectional Checklist	Between-group comparisons may be affected by baseline sociodemographic and clinical differences; regression analysis addressed DAS28 components but residual confounding remains possible.	Consecutive female outpatient sample limits applicability to broader RA populations.
Dhir V et al., 2009 [25]	Cross-sectional / JBI Analytical Cross Sectional Checklist	Controls were hospital nursing staff rather than a population-based control group, which may affect selection and group comparability.	The control source limits applicability of the RA-versus-control frequency comparison.
Ton E et al., 2012 [26]	Cross-sectional / JBI Analytical Cross Sectional Checklist	No specific internal-validity concern was identified from the reported study methods.	Tender-point subgroup sizes affect precision; tender-point count is a supportive measure, not FM diagnosis.
Nawito Z et al., 2013 [27]	Cross-sectional / JBI Analytical Cross Sectional Checklist	The FM and non-FM groups were deliberately selected, so the sample cannot estimate FM prevalence.	Female-only selected comparison and group size limit generalizability and precision.
Zammurrad S et al., 2013 [28]	Cross-sectional / JBI Analytical Cross Sectional Checklist	Non-random convenience sampling may have introduced selection bias.	No additional major precision or applicability limitation was identified from the reported study features.
da Silva Chakr RM et al., 2015 [29]	Case-control / Newcastle-Ottawa Scale (NOS)	Potential ultrasound measurement bias would depend on blinding; the study report states clinical and ultrasound scoring were blinded to FM status.	Matched selected groups and sample size limit precision and do not provide a prevalence estimate.
Ghib LJ et al., 2015 [30]	Case-control / Newcastle-Ottawa Scale (NOS)	No specific internal-validity concern was identified from the reported study methods.	Ten participants per selected group produces substantial imprecision and does not support prevalence estimation.
Mian AN et al., 2016 [31]	Cross-sectional / JBI Analytical Cross Sectional Checklist	Cross-sectional comparisons cannot establish temporality; subgroup analyses based on joint-count/tender-point criteria may be affected by residual clinical differences.	Small sample (n=47) and restriction to RA with DAS28 >2.6 limit precision and applicability to patients with lower measured disease activity.
da Silva Chakr RM et al., 2017 [32]	Cohort / Newcastle-Ottawa Scale (NOS)	Use of multiple DAS28 examiners may introduce measurement variability across visits.	Observational treatment-decision analyses remain susceptible to residual confounding; causal claims are avoided.
Salaffi F et al., 2017 [33]	Longitudinal cohort / Newcastle-Ottawa Scale (NOS)	No specific internal-validity concern was identified from the reported study methods.	Six-month follow-up restricts interpretation to short-term remission status.
Durmaz Y et al., 2021 [34]	Cross-sectional / JBI Analytical Cross Sectional Checklist	Retrospective data collection may introduce missing-data or information bias.	Single-center setting limits applicability; switching associations are non-causal.
Saitou M et al., 2022 [35]	Cross-sectional / JBI Analytical Cross Sectional Checklist	Cross-sectional associations cannot establish temporality; multivariable analyses reduce but do not eliminate residual confounding.	Only 18/240 patients were CSI screen-positive, limiting subgroup precision. CSI is a screening questionnaire for CSS-related symptom burden, not a diagnostic test for CS.
Sami M et al., 2023 [36]	Cross-sectional / JBI Analytical Cross Sectional Checklist	The FM ascertainment criterion was not reported, limiting assessment of classification validity.	The selected active-RA cohort and subgroup sizes limit generalizability.
El-Kasmi H et al., 2024 [37]	Cross-sectional / JBI Analytical Cross Sectional Checklist	Single-center cross-sectional recruitment may introduce selection bias, and regression associations remain observational.	FAST 4 and FiRST are screening instruments; positive screens are not equivalent to a clinical FM diagnosis.
Mesci N et al., 2024 [38]	Cross-sectional / JBI Analytical Cross Sectional Checklist	Cross-sectional associations cannot establish directionality between CSI symptom burden and clinical outcomes.	Small sample (n=60) limits precision; CSI ≥40 is interpreted as screen-positive symptom burden rather than diagnostic CS.
Navarini L et al., 2024 [39]	Cross-sectional / JBI Analytical Cross Sectional Checklist	Cross-sectional design limits temporal and causal interpretation of PASS-FM-DAPSA relationships.	Multicenter recruitment broadens applicability, but associations remain cross-sectional.
Kaya MN et al., 2024 [40]	Cross-sectional / JBI Analytical Cross Sectional Checklist	Cross-sectional single-center associations remain susceptible to residual confounding; logistic-regression predictors should not be interpreted causally.	A healthy-control group was not required for the within-PsA question and its absence is not treated as risk of bias. The wide CI around the PASI association limits precision.
Kieskamp SC et al., 2022 [41]	Cross-sectional analysis of GLAS cohort / JBI Analytical Cross Sectional Checklist	Cross-sectional multivariable associations cannot establish temporality; residual confounding remains possible despite adjustment.	Consecutive long-term axSpA outpatients support clinical relevance, but results may not generalize to early disease. CSI ≥40 is a screening cutoff.
Mogard E et al., 2021 [42]	Cross-sectional analysis of SPARTAKUS cohort / JBI Analytical Cross Sectional Checklist	Associations between algometry-based pain sensitivity and clinical outcomes were measured cross-sectionally and cannot establish temporal direction.	Algometry is a supportive pain-sensitivity measure rather than a diagnostic test for FM or CS; some outcome-specific analyses may have smaller effective samples.
Macfarlane GJ et al., 2019 [43]	Registry cross-sectional cluster analysis / JBI Analytical Cross Sectional Checklist	Cluster analysis was based on baseline registry data; the resulting clusters describe cross-sectional patterns and do not establish temporal relationships.	Large national sample supports precision, but cluster membership should not be interpreted as a longitudinal disease trajectory.
Wach J et al., 2016 [44]	Cross-sectional / JBI Analytical Cross Sectional Checklist	No specific internal-validity concern was identified from the reported study methods.	Small peripheral-SpA subgroup limits subgroup precision.
Gao R, 2021 [45]	Baseline comparison plus 2-month longitudinal subset / Newcastle-Ottawa Scale (NOS)	Among 107 radiographic axSpA patients eligible for longitudinal observation, 37 withdrew before the 2-month follow-up, creating substantial attrition and possible selection bias in longitudinal estimates.	Only 70 radiographic axSpA patients contributed to the short 2-month follow-up; treatment observations are non-randomized and should not be interpreted causally.
Provan SA et al., 2021 [46]	Registry cohort / Newcastle-Ottawa Scale (NOS)	Observational associations, including TNFi initiation and subsequent loss of FM classification, remain susceptible to confounding by indication, regression to the mean, and changing inflammatory activity.	FM classification was assessed at discrete follow-up visits, so interim fluctuation may be missed; associations describe classification transitions rather than treatment effects.
Kieskamp SC et al., 2025 [47]	Cross-sectional / JBI Analytical Cross Sectional Checklist	Cross-sectional structural equation modeling cannot establish temporal or causal direction among CSI, psychosocial factors, BMI, and ASDAS.	SEM estimates describe contemporaneous modeled associations and require external validation.
Aydemir B et al., 2025 [48]	Prospective observational / Newcastle-Ottawa Scale (NOS)	Repeated QST and disease-activity assessments and multivariable adjustment strengthen internal validity, but treatment initiation/switching was not randomized and residual confounding remains possible.	Twelve-week follow-up and established active RA limit inference about longer-term trajectories; QST/PPT findings are supportive pain-sensitivity measures rather than diagnostic CS.
Bertsias A et al., 2026 [49]	Prospective cohort / Newcastle-Ottawa Scale (NOS)	Prospective follow-up and multivariable analyses support the D2M association; observational treatment pathways and residual confounding limit causal interpretation of the association with FM.	Single-center targeted-therapy cohort may limit applicability; FM was recorded as a comorbidity and was not used here as a criterion-based prevalence estimate.
Carli L et al., 2026 [50]	Cross-sectional / JBI Analytical Cross Sectional Checklist	Consecutive recruitment and multivariable comparisons were reported, but cross-sectional associations do not establish temporality and shared patient-reported content may contribute to common-method bias.	CSI is a screening/symptom inventory rather than a diagnostic physiological measure; mixed PsA/AS sample and single-center recruitment affect applicability.
Chacón C et al., 2026 [51]	Cross-sectional / JBI Analytical Cross Sectional Checklist	Consecutive recruitment and multivariable modeling strengthen the analysis, but refractoriness and CSI were assessed cross-sectionally and residual confounding and common-method effects remain possible.	FM was deliberately excluded, limiting applicability to PsA with coexisting FM. Recruitment substantially overlaps in time and setting with Llamas-Ramos et al. [56], so shared outcomes were not treated as independent evidence.
Remalante-Rayco P et al., 2026 [52]	Cross-sectional analysis of longitudinal cohort / JBI Analytical Cross Sectional Checklist	Multiple imputation, bias-reduced logistic regression, prespecified covariates, and cross-validation strengthen the model; the analyzed D2M-FM relationship remains cross-sectional and cannot establish temporality.	Only 49 D2M events contributed to the principal comparison; FM was recorded as an ever comorbidity rather than as a standardized criterion-based prevalence assessment.
Akar ZA et al., 2026 [53]	Cross-sectional / JBI Analytical Cross Sectional Checklist	Validated instruments and multivariable analysis were used, but stepwise model selection and cross-sectional measurement leave residual confounding and do not establish causal direction.	PainDETECT captures a supportive neuropathic/nociplastic-like phenotype rather than diagnostic CS; single-center RA sampling limits broader generalizability.
Caglayan M et al., 2026 [54]	Cross-sectional / JBI Analytical Cross Sectional Checklist	FM was assessed with ACR 1990 criteria, but treatment exposure was not randomized and treatment-stratified associations are vulnerable to selection, confounding by indication, and reverse causation.	The SpA group was clinically heterogeneous; cross-sectional b/tsDMARD comparisons should not be interpreted as treatment effects.
Çiftci İnceoğlu S et al., 2025 [55]	Cross-sectional / JBI Analytical Cross Sectional Checklist	RA and hand-osteoarthritis groups were evaluated with validated DN4 and painDETECT instruments, but the study was a single-visit comparison and adjusted confounder analyses were not reported.	Symptomatic hand-pain recruitment limits applicability to the broader RA population; measures support neuropathic-like symptom interpretation only.
Llamas-Ramos R et al., 2026 [56]	Cross-sectional / JBI Analytical Cross Sectional Checklist	Consecutive CASPAR-classified PsA recruitment and multivariable models were reported, but cross-sectional associations cannot determine whether CSI burden precedes or follows kinesiophobia and disease impact.	Single-center sample and a generic kinesiophobia cutoff affect applicability. Recruitment overlaps in setting and time with Chacón et al. [51], so shared outcomes were not treated as independent evidence.
Petzinna SM et al., 2025 [57]	Cross-sectional / JBI Analytical Cross Sectional Checklist	Standardized DFNS QST and disease-specific classification support measurement consistency; the exploratory cross-sectional design limits temporal interpretation.	Only 20 participants per eligible disease group limits precision. SSc was outside this review scope, but RA, PsA, and axSpA results were separable and retained.
Pontes de Oliveira MNGR et al., 2025 [58]	Cross-sectional / JBI Analytical Cross Sectional Checklist	Validated CSI and painDETECT instruments were used, but convenience sampling and cross-sectional analysis leave selection and residual-confounding concerns. FM criteria were applied only among CSI-positive participants, introducing verification/ascertainment limitations for FM frequency.	Single tertiary-center RA sample limits external validity. CSI >=40 was interpreted as screen-positive symptom burden and no overall FM prevalence was inferred.
van der Kraan YM et al., 2026 [59]	Cross-sectional secondary analysis / JBI Analytical Cross Sectional Checklist	Standardized GLAS procedures and QST support measurement consistency, but the analysis was exploratory, cross-sectional, and focused on sex differences rather than causal mechanisms.	The report explicitly reanalyzed a previously published GLAS cohort and was not treated as an independent population for overlapping outcomes. CSI is not a diagnostic measure of nociplastic pain or physiological CS.

Discussion

Principal Findings

This systematic review synthesized 47 eligible primary reports across RA, PsA, axSpA, AS, and other SpA populations. A consistent observational pattern remained after the 2026 update: FM or higher central sensitization-related symptom burden was associated with higher symptom-sensitive composite disease activity scores and poorer patient-reported outcomes. The added reports strengthened, rather than simplified, the mechanistic picture. Longitudinal RA data showed that pressure pain sensitivity can improve alongside disease activity after DMARD treatment [48], while cross-sectional QST data across RA, PsA, and axSpA also identified sensory abnormalities that were not associated with inflammatory markers [57]. The evidence therefore does not support treating inflammation and altered pain processing as mutually exclusive or treating FM, CSI findings, neuropathic pain-like symptoms, PPTs, and QST as interchangeable constructs.

The clinical relevance of these associations was greatest when symptom-sensitive scores and objective inflammatory findings were discordant. In RA, several reports described higher DAS28, CDAI, or SDAI values without proportionately greater swollen joint counts, inflammatory markers, or ultrasound activity [13,27,29-31,36,38,53,57,58]. Across disease groups, FM or higher CSI symptom burden was also associated with greater pain, impaired function, sleep or mood symptoms, and poorer quality of life [15,19,21,24,25,35,37-41,43-47,50,51,53,56,58,59]. Difficult-to-manage or treatment-refractory phenotypes were additionally associated with FM or higher CSI burden in recent axSpA and PsA reports [49-52]. These findings are observational and do not establish that altered pain processing is the sole cause of persistent symptoms, elevated disease activity scores, or treatment difficulty.

Prevalence Findings

Reported frequencies varied substantially by disease group, clinical setting, case definition, screening instrument, and patient selection. The update added criterion-based FM estimates in RA and SpA using ACR 1990 criteria [54], a lower 2016-criteria FM frequency in one PsA cohort [56], and additional CSI screen-positive findings ranging from 35.1% in a PsA cohort excluding FM to 59.29% in a tertiary-care RA cohort [50,51,58]. Criterion-based FM, FiRST or FAST 4 screening-positive findings, CSI screen-positive symptom burden, and supportive pain phenotypes were therefore kept separate. Selected comparison groups were not interpreted as prevalence samples, and no overall prevalence was inferred across non-equivalent constructs.

This distinction is clinically important because the instruments answer different questions. ACR FM criteria and Fibromyalgia Survey Diagnostic Criteria classify syndrome-level FM; FiRST and FAST 4 are screening instruments; the Central Sensitization Inventory is a symptom inventory rather than a diagnostic test for central sensitization; and pressure pain thresholds, quantitative sensory testing, painDETECT, and DN4 provide purpose-specific supportive information on pain sensitivity or neuropathic-like symptoms [13,15,18,20-22,35,38,40-42,47,48,50,51,53,55-59]. Recent reports themselves emphasized these construct limits, particularly the distinction between CSI symptom burden and physiological or diagnostic evidence of central sensitization [50,59].

Interpretation of Disease Activity and Objective Inflammation

Across disease groups, FM or higher central sensitization-related symptom burden was repeatedly associated with higher composite disease activity scores, particularly measures containing pain-sensitive or patient-reported components [13-18,21,23-29,34,38-47,50,51,53,56,58,59]. In RA, DAS28-derived measures, such as DAS28-P, illustrate how the relative contribution of patient-reported components can increase when FM or pain sensitivity is present [13]. Aydemir et al. showed that reductions in DAS28-CRP were associated with increased PPTs after DMARD treatment, whereas CRP change itself was not associated with PPT change [48]. Akar et al. similarly found stronger correlations of painDETECT with pain and DAS28 than with CRP or ESR [53]. In PsA and axSpA, DAPSA, CPDAI, BASDAI, ASDAS, and related indices incorporate symptom-sensitive domains and should be interpreted in the context of the construct being measured.

The clearest evidence for clinical-objective discordance remained the RA ultrasound literature: patients with FM or fibromyalgic clinical features often had higher clinical disease activity scores while ultrasound synovitis or power Doppler activity was similar or lower than in comparison groups [29-31,36]. Other reports described higher tender joint count or patient global assessment with comparable swollen joint count, ESR, or CRP [27,38]. The update added complementary evidence rather than a uniform pattern: Petzinna et al. observed standardized QST abnormalities across RA, PsA, and axSpA without association with inflammatory markers [57], while Aydemir et al. showed partial coupling between improvement in disease activity and pressure pain sensitivity [48]. These findings do not show that inflammation is absent; they demonstrate that clinical scores and pain-processing measures may capture overlapping but non-identical processes.

FM and active inflammation are not mutually exclusive. Dhir et al. [25], for example, reported higher tender and swollen joint counts among patients with FM. Therefore, discordant symptom burden should prompt reassessment rather than automatic attribution to either inflammation or centralized pain. Clinical examination, CRP or ESR, and imaging when indicated can help reassess inflammatory activity, while structural, mechanical, FM-related, sleep, mood, and neuropathic-like contributors require separate purpose-specific assessment.

Treatment and Treat-to-Target Implications

Treatment-related evidence was observational or exploratory and should not be interpreted causally. FM or higher central sensitization-related symptom burden was associated with differences in remission classification, minimal disease activity, treatment retention, biologic switching, treatment-decision patterns, or difficult-to-manage/refractory phenotypes in several reports [14,23,32-34,39,46,49-52,54]. The recent D2M axSpA reports identified FM as an independently associated factor [49,52], while Chacón et al. found a strong cross-sectional association between CSI screen positivity and therapeutic refractoriness in PsA [51]. These associations may reflect symptom burden, inflammatory activity, treatment history, patient expectations, confounding by indication, or other factors and do not establish that FM or central sensitization causes treatment failure, inappropriate escalation, or undertreatment.

Importantly, higher symptom burden did not uniformly imply lack of anti-inflammatory response. Macfarlane et al. [17] reported higher baseline BASDAI and poorer quality of life among participants meeting FM criteria but no significant difference in ASAS20 response at 12 months after TNF inhibitor initiation. In Provan et al. [46], FM classification changed over time, and TNF inhibitor initiation was associated with subsequent loss of FM classification, but causal inference is not possible. Caglayan et al. found lower FM frequency among b/tsDMARD-treated than untreated participants, particularly within RA, but the cross-sectional design cannot establish a treatment effect [54]. Aydemir et al. observed improvement in PPTs following DMARD changes while temporal summation and conditioned pain modulation did not significantly change [48]. Together, these findings support separating inflammatory response, symptom burden, pain sensitivity, and treatment persistence rather than interpreting any single construct as treatment success or failure.

These observations are relevant to treat-to-target practice because symptom-sensitive composite scores may remain elevated even when inflammatory activity is limited. The EULAR difficult-to-treat RA framework similarly emphasizes establishing whether inflammatory activity is present before adjusting disease-modifying therapy and considering comorbid contributors, including FM, when persistent symptoms are not fully explained by inflammation [60]. In this review, the practical implication is not to replace validated disease activity measures, but to interpret them alongside objective inflammatory assessment and the broader clinical context.

Strengths, Limitations, and Certainty of Interpretation

This review has several strengths. It synthesized evidence across the inflammatory arthritis and SpA populations represented in the 47 eligible reports; separated criterion-based FM, screening-positive findings, CSI symptom burden, and supportive pain phenotypes; integrated clinical scores with patient-reported, sensory, and objective inflammatory outcomes; and used design-appropriate RoB tools. The July 28, 2026 update used a broader two-concept PubMed/MEDLINE strategy without a mandatory outcome block and added 12 eligible reports. Ultrasound and power Doppler studies remained particularly informative for clinical-objective discordance, while newer longitudinal and standardized QST studies broadened the evidence on pain sensitivity [29-31,36,48,57].

Several limitations should also be acknowledged. The protocol was not prospectively registered in PROSPERO. The review was restricted to English-language reports, which may introduce language bias. For the original search, source-specific retrieval counts, exact interface-specific search dates, database-platform details, and line-by-line executable histories were not retained separately after records were merged; these details could not be reconstructed reliably. The July 28, 2026 PubMed/MEDLINE update was documented with its exact executable query and screening outcome, but it does not retrospectively remove limitations of the historical search documentation. Some reports arose from overlapping or related cohorts, including potentially overlapping Salamanca PsA reports and secondary GLAS analyses [51,56,59]; these were retained only for distinct report-level contributions and were not treated as independent evidence for the same outcome. After structured assessment of quantitative-synthesis feasibility, meta-analysis was not undertaken because sufficiently comparable independent subsets with compatible definitions, denominators, effect measures, and numerical data were not consistently available. Most evidence was cross-sectional, limiting temporal and causal inference, and no formal GRADE or other certainty-of-evidence assessment was performed.

RoB findings were interpreted within the structure of the design-appropriate appraisal tool rather than converted into a common severity scale. Bias-relevant concerns included non-random sampling, incomplete recruitment reporting, residual confounding, measurement limitations, and attrition where applicable; small sample size, single-center recruitment, and restricted subgroup size were treated primarily as precision or applicability limitations unless they created a specific internal-validity concern. Patient-reported outcomes were not considered inherently problematic when the construct itself was patient-centered, and validated screening instruments were interpreted according to their intended purpose rather than treated as diagnostic substitutes.

Because no formal certainty assessment was performed, GRADE-style certainty categories were not assigned. Observational evidence suggests associations between FM or central sensitization-related symptom burden and higher composite disease activity scores and poorer patient-reported outcomes, but the magnitude and causal implications remain uncertain. Apparent consistency across reports may partly reflect shared patient-reported components, common-method bias, and overlapping cohorts. Objective inflammatory-marker, imaging, longitudinal QST, and standardized sensory-testing reports strengthen the observation that high symptom-sensitive scores do not always correspond to proportionately greater inflammation, while also showing that pain sensitivity may change alongside inflammatory disease activity in some settings [29-31,36,48,53,57]. The evidence does not demonstrate that identifying FM or central sensitization improves treatment decisions or reduces misclassification.

Clinical and Research Implications

When persistent symptom burden appears disproportionate to objective inflammatory findings, assessment should be stepwise. Inflammatory activity should first be reassessed using clinical examination and, where appropriate, CRP, ESR, and imaging. Structural or mechanical contributors should then be considered. FM should be evaluated using accepted criteria, while FiRST or FAST 4 may be used as screening instruments rather than diagnostic substitutes. Sleep, mood, function, widespread pain, and neuropathic-like symptoms can be assessed with purpose-specific instruments. The Central Sensitization Inventory may characterize central sensitization-related symptom burden, while painDETECT or DN4 may screen neuropathic-like features; no single questionnaire, laboratory marker, or imaging test establishes the underlying pain mechanism. Inflammation, FM, mechanical pain, and other pain mechanisms may coexist.

Future research should prioritize longitudinal and interventional studies that distinguish inflammatory activity from FM, central sensitization-related symptom burden, and other pain mechanisms; evaluate how these features change over time and with treatment; compare assessment tools directly; and test whether structured assessment pathways improve disease-activity interpretation, patient-centered outcomes, or treatment decisions. Reports from shared registries or cohorts should explicitly identify participant overlap, and future syntheses would benefit from harmonized definitions and outcome reporting that allow independent quantitative pooling. Until such evidence is available, these findings support a more complete clinical interpretation rather than a new treatment algorithm.

## Conclusions

FM and central sensitization-related findings were frequently reported and clinically relevant across the RA, PsA, and SpA populations included in this review. Across 47 eligible primary reports, they were associated with higher symptom-sensitive composite disease activity scores, greater patient-reported symptom burden, impaired function, poorer sleep, mood symptoms, and reduced quality of life. Observational evidence also linked these features with difficult-to-manage or treatment-refractory phenotypes and treatment-related classifications, but did not establish causal treatment effects. Associations were particularly evident in composite indices that include pain-sensitive and patient-reported components.

Persistently elevated disease activity scores should not automatically be interpreted as uncontrolled inflammation, particularly when objective inflammatory findings are limited or discordant. Clinical assessment should be stepwise: inflammatory activity should first be reassessed using clinical examination and CRP or ESR, with imaging when appropriate; structural, mechanical, and other non-inflammatory contributors should then be considered; and fibromyalgia, central sensitization-related symptom burden, sleep, mood, and neuropathic pain-like features should be evaluated using purpose-specific criteria or instruments. No single questionnaire, laboratory marker, or imaging test establishes the underlying pain mechanism, and fibromyalgia or central sensitization-related symptoms may coexist with active inflammation. Integrating these findings may support a more complete interpretation of persistent symptoms.
